# Discovery of potent and selective inhibitors of human NLRP3 with a novel mechanism of action

**DOI:** 10.1084/jem.20242403

**Published:** 2025-09-02

**Authors:** Kevin Wilhelmsen, Aditi Deshpande, Sarah Tronnes, Maitriyee Mahanta, Matthew Banicki, Mary Cochran, Samantha Cowdin, Kristen Fortney, George Hartman, Robert E. Hughes, Rusty Montgomery, Claudia P. Portillo, Paul Rubin, Taiz Salazar, Yan Wang, Shijun Yan, Barry A. Morgan, Assem Duisembekova, Romane Riou, Michael Marleaux, Inga V. Hochheiser, Hannes Buthmann, Dominic Ferber, Jane Torp, Wei Wang, Melanie Cranston, Chloe M. McKee, Thea J. Mawhinney, Emma C. McKay, Fehime K. Eroglu, Jasmin Kümmerle-Deschner, Alexander N.R. Weber, Bénédicte F. Py, Matthias Geyer, Rebecca C. Coll

**Affiliations:** 1 BioAge Labs, Emeryville, CA, USA; 2 HitGen Pharmaceuticals Inc., Houston, TX, USA; 3 CIRI, Centre International de Recherche en Infectiologie, Univ Lyon, Inserm, U1111, Université Claude Bernard Lyon 1, CNRS, UMR5308, ENS de Lyon, Lyon, France; 4 Institute of Structural Biology, University of Bonn, Bonn, Germany; 5 Wellcome-Wolfson Institute for Experimental Medicine, Queen’s University Belfast, Belfast, UK; 6 Department of Innate Immunity, Institute of Immunology, Tübingen, Germany; 7 Department of Pediatrics I, Pediatric Rheumatology and Autoinflammation Reference Center, University Hospital Tübingen, Tübingen, Germany; 8 Cluster of Excellence 2180 “Image-guided and Functionally Instructed Tumor Therapies” and Cluster of Excellence 2124 “Controlling Microbes to Fight Infection”, University of Tübingen, Tübingen, Germany

## Abstract

The NLRP3 inflammasome is an intracellular protein complex that causes inflammation via the release of IL-1β and pyroptosis. NLRP3 activation is associated with many age-related inflammatory diseases, and NLRP3 inhibition is a promising therapeutic strategy. We previously performed a DNA-encoded library screen to identify novel NLRP3-binding molecules. Herein we describe the characterization of BAL-0028 as a potent and specific inhibitor of NLRP3 signaling. Notably, BAL-0028 is a poor inhibitor of mouse NLRP3 but inhibits human and primate NLRP3 with nanomolar potency. Using cellular and biochemical analyses, we demonstrate that BAL-0028 binds to the NLRP3 NACHT domain at a site that is distinct from the MCC950-binding pocket. Using humanized NLRP3 mice, we show that a derivative of BAL-0028, BAL-0598, inhibits NLRP3 activation *in vivo* in a peritonitis model. Finally, we demonstrate that both BAL-0028 and BAL-0598 inhibit select hyperactive NLRP3 mutations associated with autoinflammatory diseases more potently than MCC950. BAL-0028 and BAL-0598 thus represent a new modality for NLRP3 inhibition in inflammatory diseases.

## Teaser

NLRP3 is a target for anti-inflammatory therapies and can be inhibited by the tool compound MCC950. We describe the characterization of new small-molecule inhibitors of NLRP3, BAL-0028 and BAL-0598, that have a distinct mechanism of action and binding site.

## Introduction

Inflammation is associated with many age-related diseases, including neurodegenerative conditions, cancer, and metabolic and cardiovascular diseases (Franceschi et al., 2018; Ransohoff, 2016). Limiting inflammation may thus be an effective therapeutic strategy to reduce or delay age-related diseases (Figueira et al., 2016). Inflammasomes are protein complexes that have emerged as central mediators of inflammation, as they control the production of the pro-inflammatory cytokines IL-1β and IL-18 and lytic inflammatory cell death known as pyroptosis (Broz and Dixit, 2016). Inflammasomes are formed by a pattern recognition receptor or sensor molecule that interacts with the adapter molecule apoptosis-associated speck-like protein containing a caspase activation and recruitment domain (ASC). ASC oligomerizes and provides a platform for the autocatalytic activation of the zymogen protease caspase-1. Active caspase-1 cleaves pro–IL-1β and IL-18 into their active secreted forms and mediates pyroptosis via cleavage of gasdermin D (Fu et al., 2024).

Amongst the known inflammasome sensors, the NACHT, LRR, and PYD–containing protein 3 (NLRP3) has emerged as a key mediator of pathogenic inflammation in many inflammatory diseases. NLRP3 can be activated by a broad range of molecules and processes and is regarded as a sensor of the disruption of cellular homeostasis (Akbal et al., 2022). Disease-related molecules such as β-amyloid, α-synuclein, and monosodium urate (MSU) crystals have been shown to trigger NLRP3 activation. NLRP3-deficient mice are correspondingly protected in models of Alzheimer’s disease, Parkinson’s disease, and gout. There is also a group of rare genetic diseases caused by gain-of-function mutations in *NLRP3* called cryopyrin-associated periodic syndromes or *NLRP3*-associated autoinflammatory diseases (NLRP3-AID). NLRP3 inhibition is thus a promising anti-inflammatory therapeutic strategy (Coll et al., 2022; Mangan et al., 2018).

Currently available therapies to inhibit the inflammasome pathway are limited to biologics such as anakinra (IL-1 receptor antagonist) and canakinumab (anti–IL-1β) (Coll, 2023). However, specific NLRP3 inhibition could have several advantages over these, including preventing IL-18 release and pyroptosis, while maintaining inflammation driven by other inflammasomes like NLRP1 (NACHT, LRR, and PYD-containing protein 1) and NLRC4 (NLR family CARD-containing protein 4) that may be important for host defense (Barnett et al., 2023). In addition, small-molecule inhibitors have improved blood–brain barrier crossing, which would be critical for blocking neuroinflammation (Xiong et al., 2021).

Numerous small-molecule NLRP3 inhibitors have been described, including the sulfonylurea MCC950 (aka CRID3, CP-456,773). MCC950 was developed based on early observations from cell-based screens that identified diarylsulfonylureas such as glyburide that could inhibit NLRP3 signaling (Laliberte et al., 2003; Lamkanfi et al., 2009; Perregaux et al., 2001). MCC950 is the most widely used tool molecule to study NLRP3 inhibition in cells and in animal models of disease (Coll et al., 2015; Corcoran et al., 2021). MCC950 is a specific non-covalent inhibitor that blocks the ATPase activity of NLRP3 (Coll et al., 2019; Tapia-Abellán et al., 2019; Vande Walle et al., 2019). Its binding site has been resolved at molecular resolution with two reports of NLRP3-MCC950 cryo-EM structures (Hochheiser et al., 2022b; Ohto et al., 2022) and a crystal structure of NLRP3 with an MCC950 analog (Dekker et al., 2021). Notably, the majority of NLRP3 inhibitors that have advanced to early stage clinical trials are sulfonylureas or MCC950 derivatives (Vande Walle and Lamkanfi, 2024).

Several other direct NLRP3 inhibitors with different chemistries and mechanisms of action have been described, such as CY-09, which prevents ATP binding, and tranilast, which disrupts NLRP3 oligomerization (Coll et al., 2022; Huang et al., 2018; Jiang et al., 2017). However, many of these compounds have off-target effects, and indeed MCC950 has also been shown to inhibit carbonic anhydrase 2 (Coll et al., 2022; Kennedy et al., 2021; Vande Walle et al., 2024). Importantly, in studies of NLRP3-AID mouse models and patient cells, it has been observed that MCC950 cannot effectively inhibit NLRP3 activation by certain mutations (Cosson et al., 2024; Vande Walle et al., 2019). There is therefore a need to identify specific NLRP3 inhibitors with novel mechanisms of action that can offer an alternative to MCC950-derived compounds. Herein we describe BAL-0028 and BAL-0598 as specific inhibitors of NLRP3 with a mechanism that is distinct from MCC950. Remarkably, we find that BAL-0028 and BAL-0598 potently inhibit primate NLRP3 but have significantly reduced potency for NLRP3 from other mammals, highlighting the importance of drug development efforts focused on human proteins.

## Results

**Figure 1.**
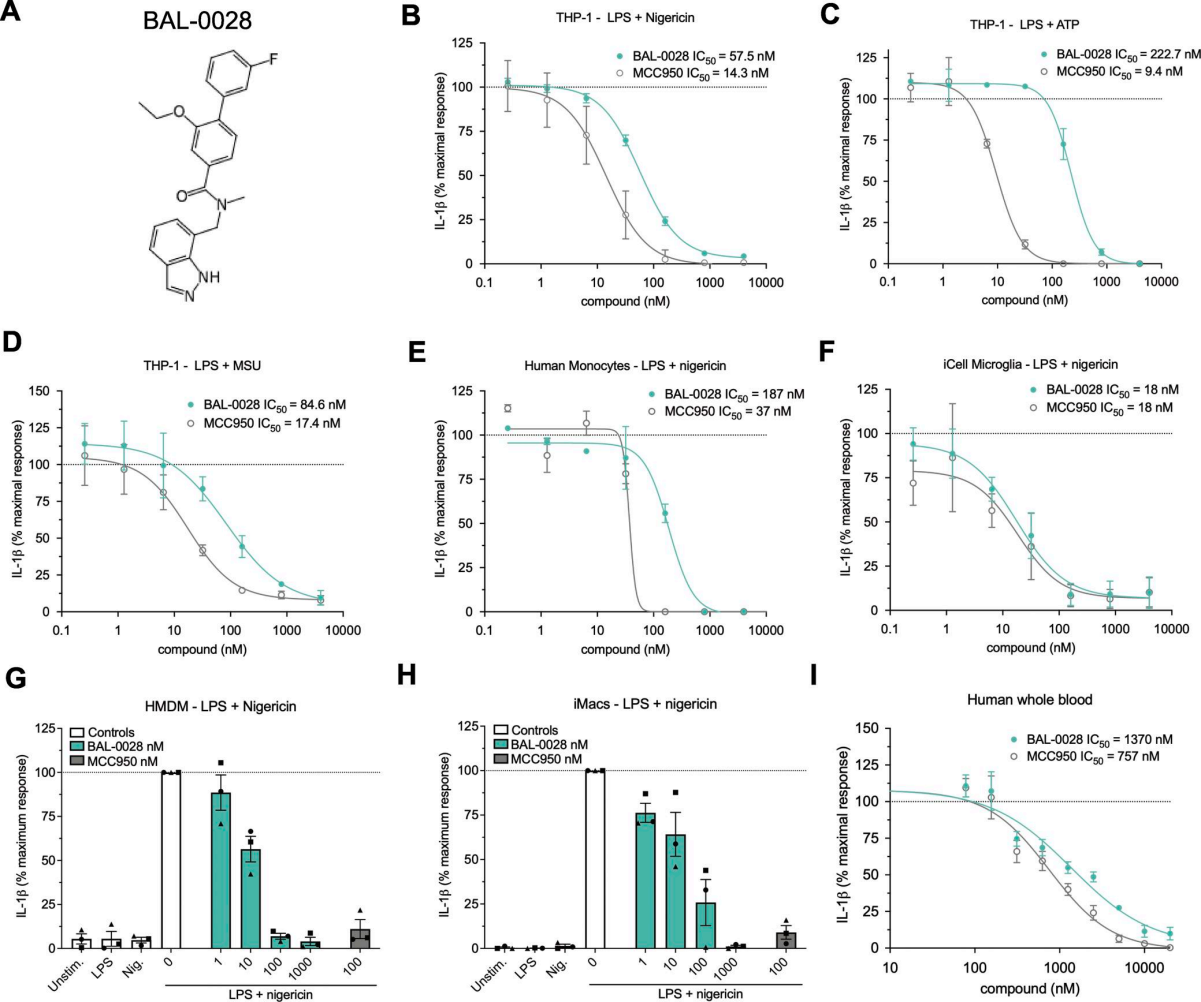
**BAL-0028 is a potent inhibitor of NLRP3 signaling in multiple human cell types. (A)** Structure of BAL-0028. **(B–D)** Comparison of BAL-0028 and MCC950 in IL-1β release assays from PMA-differentiated THP-1 cells stimulated with LPS and (B) nigericin, (C) ATP, or (D) MSU. **(E–I)** Comparison of BAL-0028 and MCC950 in IL-1β release assays from LPS and nigericin-stimulated human monocytes (E), iCell microglia (F), HMDM (G), iMacs (H), and human whole blood (I). **(B–F and I)** Graph symbols show average IL-1β values relative to vehicle control ± SEM from independent experiments performed in triplicate; the IC_50_ curve was fitted by nonlinear regression analysis. **(G and H)** Graph symbols show average values relative to vehicle control from independent experiments (indicated by different symbols) performed in triplicate ± SEM. Compounds are shown in nanomolar (nM) concentrations. *N* = 73 for BAL-0028 and *N* = 29 for MCC950 (B), *N* = 2 (C, D, and E), *N* = 3 (F and H), *N* = 3 donors (G), and *N* = 4 donors (I).

We previously used a DNA-encoded chemical library (DEL) screen to identify novel NLRP3-binding chemical structures with strong potential for optimization to clinical candidates, specifically for the treatment of neurological diseases (Hartman et al., 2024). The DEL screen used a protein construct of human NLRP3 lacking the amino-terminal PYD, and this construct (maltose-binding protein [MBP]-ΔNLRP3-HIS) was used to screen against small-molecule libraries encompassing >500 billion compounds. Our DEL screening conditions were optimized to enhance the stability of NLRP3 with retention of ATPase activity and used several NLRP3 inhibitors to block their binding sites and thereby enhance the ligand analysis efforts. This resulted in the identification of a series of indazole small-molecule NLRP3 binders. A high-affinity (*K*_D_ range 104–123 nM) lead compound from this study, BAL-0028 (Fig. 1 A), was selected for further characterization in cell-based assays (Hartman et al., 2024). We first examined BAL-0028 in NLRP3 signaling assays using THP-1 macrophage-like cells. Pre-treatment with BAL-0028 potently inhibits NLRP3-dependent IL-1β release from THP-1s simulated with LPS and the pore-forming toxin nigericin, with an IC_50_ value of 57.5 nM. In the same assay, the IC_50_ of MCC950 is 14.3 nM, which is consistent with previous reports (Clénet et al., 2023; Teske et al., 2024) (Fig. 1 B). NLRP3 is activated by many molecules, including damage-associated molecular patterns released during sterile inflammation, such as ATP and MSU crystals (Kapetanovic et al., 2015; Mangan et al., 2018). BAL-0028 also inhibits IL-1β release triggered by ATP and MSU with IC_50_s in the nanomolar range (Fig. 1, C and D). This confirms the ability of BAL-0028 to block NLRP3-dependent signaling by multiple stimuli in THP-1s.

**Figure S1.**
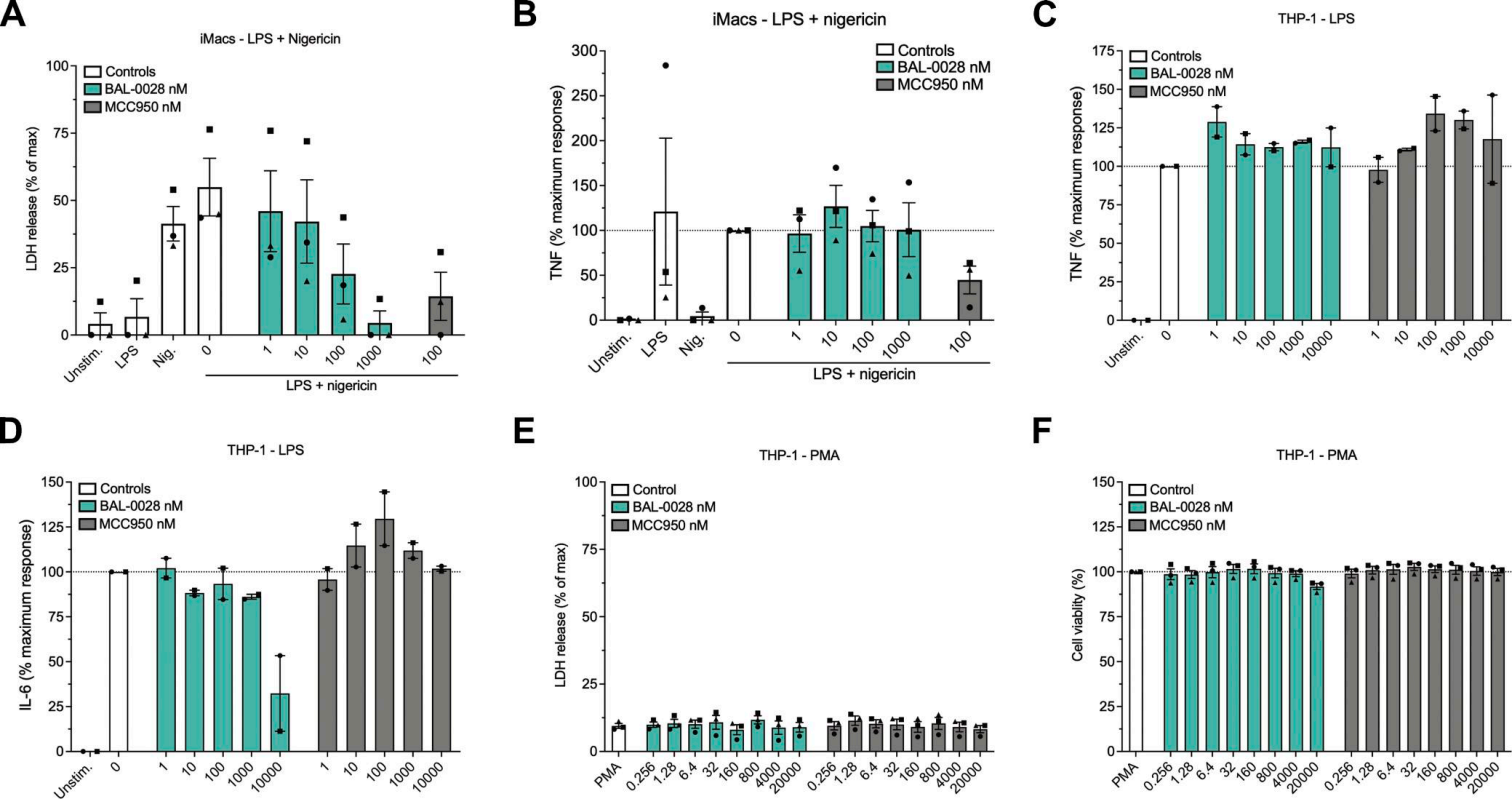
**BAL-0028 does not inhibit inflammasome-independent cytokine release and is not cytotoxic** **.** Related to Fig. 1. **(A and B)** Effect of BAL-0028 and MCC950 on LDH release (A) and TNF release (B) from LPS- and nigericin-stimulated iMacs. **(C and D)** Comparison of BAL-0028 and MCC950 pre-treatment in PMA-differentiated THP-1s on LPS-induced secretion of (C) TNF and (D) IL-6. **(E and F)** Effect of BAL-0028 and MCC950 treatment in PMA-differentiated THP-1s on (E) cytotoxicity (LDH release) (F) and cell viability (CellTiter-Blue assay). **(A–F)** Bar chart symbols show average values relative to vehicle control from independent experiments (indicated by different symbols) performed in triplicate ± SEM. *N* = 3 (A, B, E, and F) and *N* = 2 (C and D).

BAL-0028 was further evaluated in a range of more physiologically relevant human cell types. BAL-0028 consistently inhibits nigericin-induced IL-1β release in primary monocytes (Fig. 1 E) and induced pluripotent stem cell (iPSC)-derived microglia (iCell microglia) (Fig. 1 F). The IC_50_ of BAL-0028 is generally higher than MCC950 (Fig. 1, B–E); however, for iCell microglia, the compounds are equipotent (Fig. 1 F). Having established the potency of BAL-0028, we tested it in primary human monocyte-derived macrophages (HMDM) and iPSC-derived macrophages (iMacs). BAL-0028 inhibited nigericin-induced IL-1β release in HMDM and iMacs in the nanomolar range (Fig. 1, G and H). In addition, we examine lactate dehydrogenase (LDH) release as a measure of pyroptotic cell death induced by NLRP3. LDH is dose dependently inhibited by BAL-0028 in iMacs (Fig. S1 A). Together, these data demonstrate that BAL-0028 is a potent inhibitor of NLRP3 signaling in multiple human cell types. Stimulation with LPS is required to induce both transcriptional and posttranslational priming of the NLRP3 inflammasome (McKee and Coll, 2020; O’Keefe et al., 2024). To examine the effects of BAL-0028 on LPS signaling, we measured the release of inflammasome-independent inflammatory cytokines TNF and IL-6. BAL-0028 does not reduce TNF secretion from iMacs in NLRP3 assays (Fig. S1 B). Pre-treatment with BAL-0028 does not block LPS-induced TNF secretion, although there was some reduction in IL-6 release at the highest concentration of BAL-0028 (10 mM) (Fig. S1, C and D). These data demonstrate that BAL-0028 does not appreciably interfere with LPS signaling. We also confirmed that BAL-0028 is not cytotoxic, as it does not increase LDH release or reduce cell viability in THP-1s (Fig. S1, E and F). Lastly, we compared BAL-0028 and MCC950 in a whole blood NLRP3 assay. As expected, there was a decrease in potency of both BAL-0028 and MCC950 due to plasma protein binding, but both compounds effectively inhibited IL-1β release (Fig. 1 I). This confirms that BAL-0028 is a potent inhibitor of human NLRP3 in a clinically relevant assay.

**Figure 2.**
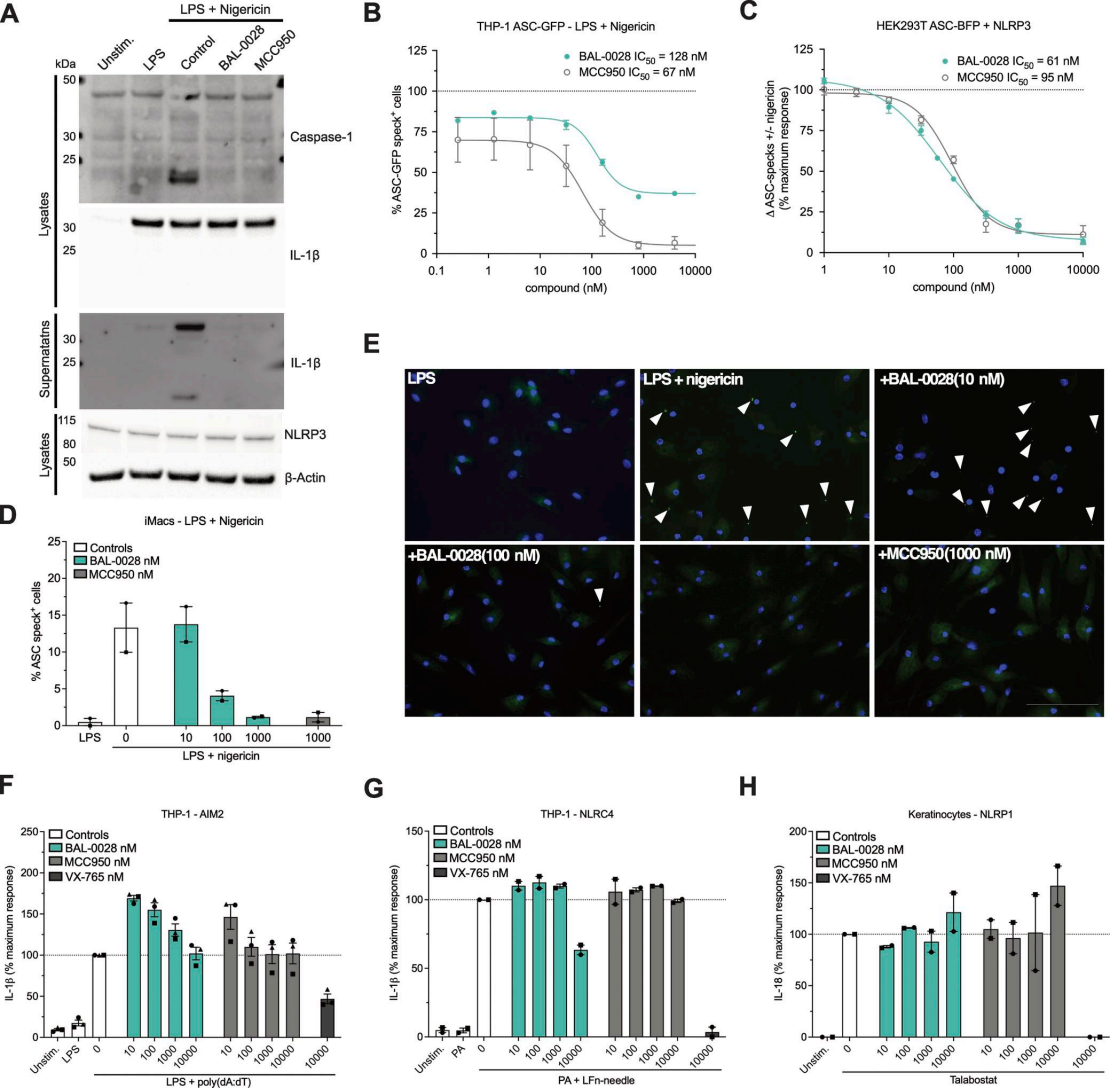
**BAL-0028 specifically inhibits NLRP3 inflammasome formation. (A)** Western blot for caspase-1 and IL-1β cleavage and NLRP3 expression from PMA-differentiated THP-1 cells stimulated with LPS and nigericin in the presence of BAL-0028 or MCC950 (both 500 nM). **(B)** Comparison of BAL-0028 and MCC950 effects on ASC speck formation assessed by fluorescence microscopy in PMA-differentiated THP-1 ASC-GFP cells stimulated with LPS and nigericin. **(C)** Comparison of BAL-0028 and MCC950 effects on ASC speck formation assessed by flow cytometry in HEK293T ASC-BFP cells transfected with human NLRP3 and stimulated with nigericin. **(D and E)** Effect of BAL-0028 and MCC950 on ASC speck formation assessed by fluorescence microscopy using an anti-ASC antibody in iMacs. **(F and G)** Effects of BAL-0028, MCC950, and VX-765 on IL-1β release from PMA-differentiated THP-1 cells stimulated with (F) LPS and transfected with poly(dA:dT) or (G) protective antigen (PA) and Lfn-needle protein. **(H)** Effects of BAL-0028, MCC950, and VX-765 on IL-18 release from human keratinocytes stimulated with talabostat. **(A)** Representative blots from *N* = 2 independent experiments. **(B)** Average ± SEM % ASC-GFP speck-positive cells from *N* = 2 independent experiments performed in triplicate. **(C)** Average ± SEM change in nigericin-induced ASC specks normalized to cells without compound treatment from *N* = 3–4 independent experiments. **(D and F–H)** Graph symbols show average values relative to vehicle control from independent experiments performed in triplicate (indicated by different symbols) ± SEM. *N* = 2 (D, G, and H) and *N* = 3 (F). **(E)** Representative images from D, scale bar is 100 μM. Source data are available for this figure: SourceData F2.

We next examined the effects of BAL-0028 on signaling events upstream of IL-1β secretion and pyroptosis. Upon NLRP3 inflammasome activation, pro–IL-1β is cleaved into its active p17 form by caspase-1 (Broz and Dixit, 2016). Through western blotting, we confirmed that both BAL-0028 and MCC950 inhibited pro–IL-1β processing and caspase-1 activation as measured by the appearance of the p20 auto-processed form of caspase-1 (Boucher et al., 2018) (Fig. 2 A). BAL-0028 did not affect the expression of pro–IL-1β or NLRP3, again suggesting NLRP3 priming by LPS is unaffected by BAL-0028 (Fig. 2 A). To examine whether NLRP3 inflammasome formation is inhibited by BAL-0028, we measured ASC speck formation by fluorescence microscopy. In THP-1 cells stably expressing GFP-tagged ASC, nigericin-induced ASC speck formation is dose dependently inhibited by both BAL-0028 and MCC950 (Fig. 2 B). We next used HEK293T cells expressing BlueFP-ASC and doxycycline-inducible NLRP3 to monitor ASC specks by flow cytometry. In these cells, nigericin stimulation triggers a significant increase in ASC specks, which is dose dependently inhibited by both BAL-0028 and MCC950 (Fig. 2 C). In addition, in iMacs, nigericin-induced ASC speck formation was also potently blocked by BAL-0028 (Fig. 2, D and E). These data confirm that BAL-0028 inhibits the formation of the NLRP3 inflammasome.

**Figure S2.**
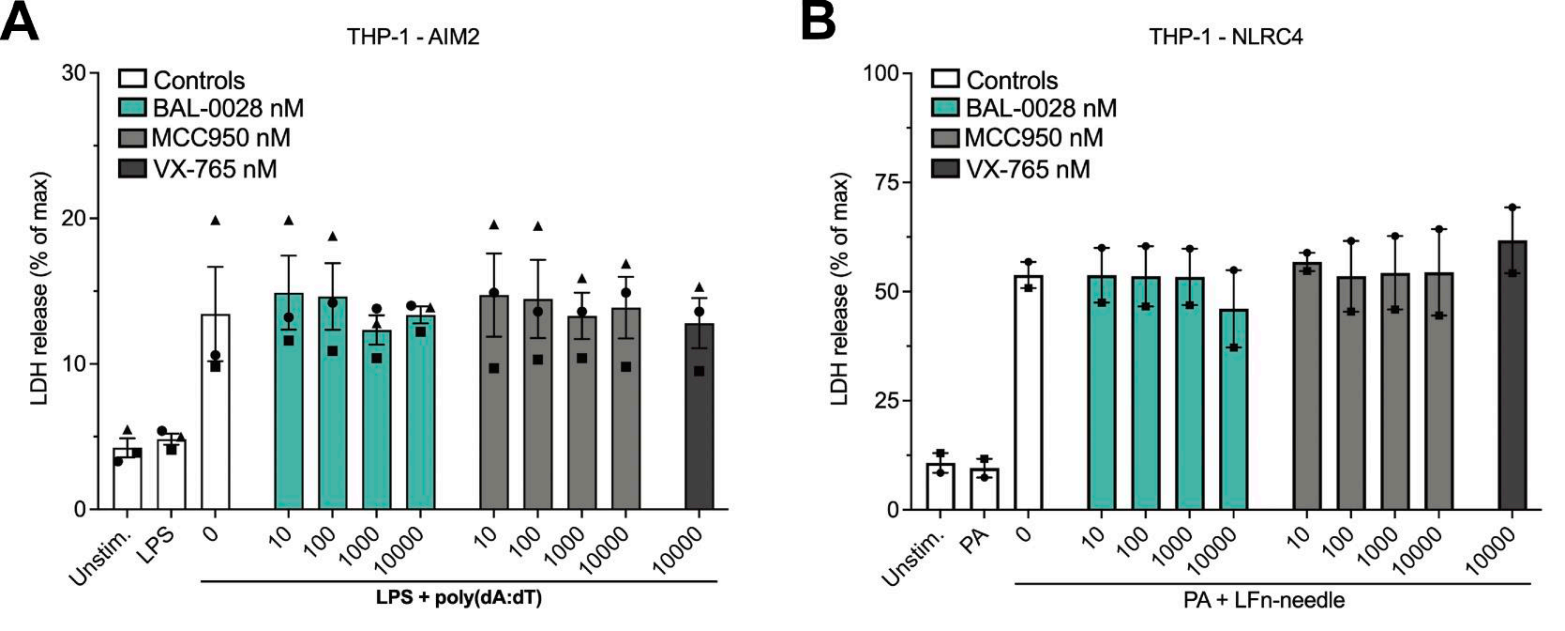
**BAL-0028 does not inhibit AIM2- or NAIP/NLRC4-dependent pyroptosis** **.** Related to Fig. 2. **(A and B)** Effects of BAL-0028, MCC950, and VX-765 on LDH release from PMA-differentiated THP-1 cells stimulated with (A) LPS and transfected with poly(dA:dT) or (B) protective antigen (PA) and Lfn-needle protein. Graph symbols show average values from independent experiments performed in triplicate (indicated by different symbols) ± SEM. *N* = 3 (A) and *N* = 2 (B).

**Figure 3.**
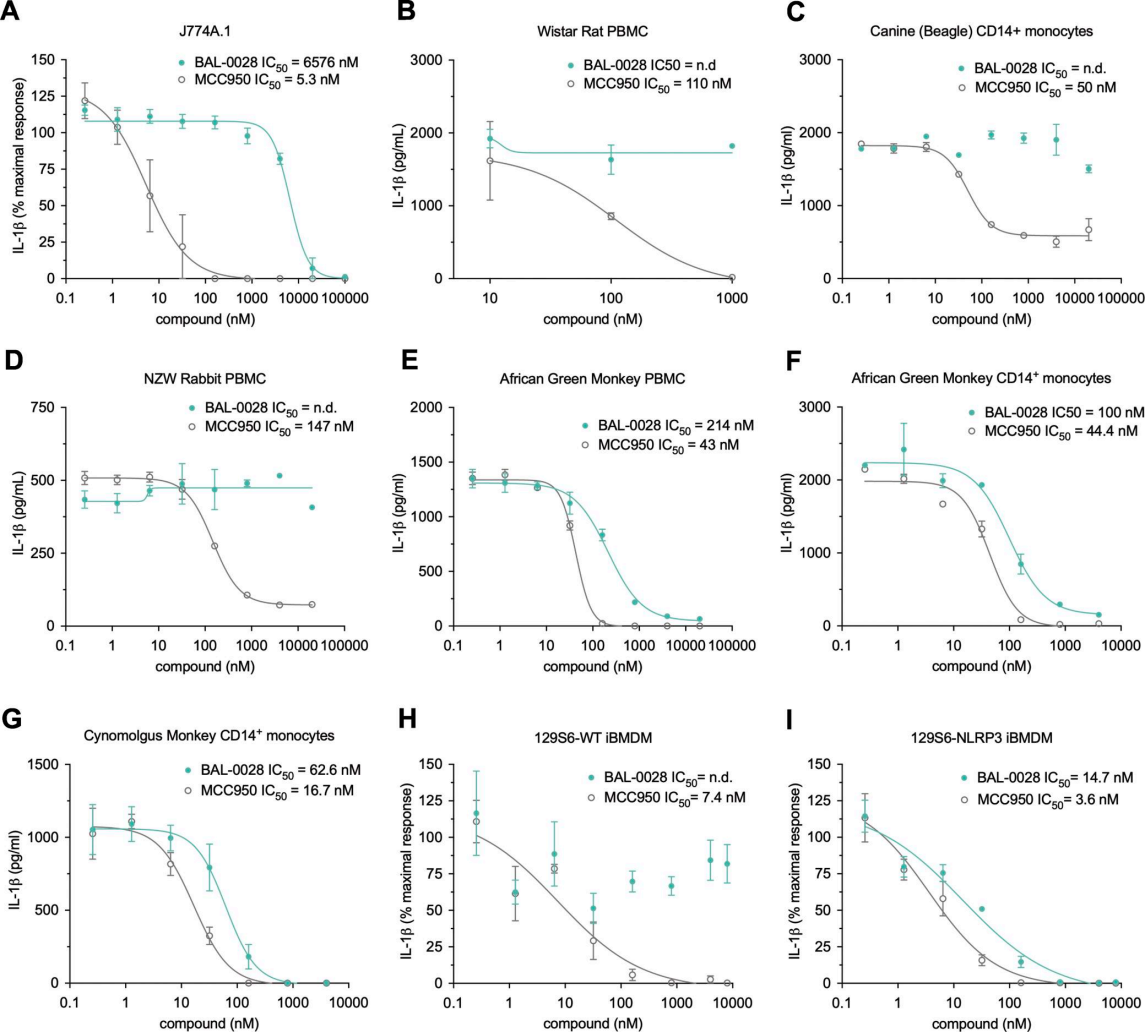
**BAL-0028 inhibits primate NLRP3 but is a poor inhibitor of NLRP3 from other mammals.** Comparison of BAL-0028 and MCC950 in IL-1β release assays from cells stimulated with LPS and nigericin. **(A–I)** J774A.1 mouse macrophage cell line (A), Wistar rat PBMCs (B), Beagle CD14^+^ monocytes (C), New Zealand white rabbit PBMCs (D), African green monkey (*C. sabaeus*) PBMCs (E) and CD14^+^ monocytes (F), cynomolgus monkey (*M. fascicularis*) CD14^+^ monocytes (G), WT 129S6 iBMDM (H), and 129S6-human promoter *NLRP3* iBMDM (I). **(A, H, and I)** Graph symbols show average IL-1β values relative to vehicle control ± SEM from *N* = 3 independent experiments performed in triplicate. **(B–G)** Graph symbols show average IL-1β values relative to vehicle control ± SD from one experiment performed in duplicate (C, D, and F) or triplicate (B, E, and G). IC_50_ curves were fitted by nonlinear regression analysis.

To confirm the specificity of BAL-0028 for NLRP3, we assessed the effects of BAL-0028 on other inflammasome pathways. Transfection of the synthetic double-stranded DNA poly(dA:dT) in THP-1s induces IL-1β release via the dsDNA sensor AIM2 (Fernandes-Alnemri et al., 2009; Hornung et al., 2009). In this AIM2 assay, BAL-0028 and MCC950 did not reduce IL-1β release relative to the vehicle control, while the caspase-1 inhibitor VX-765 (Wannamaker et al., 2007) attenuated IL-1β release (Fig. 2 F). The NAIP/NLRC4 inflammasome senses bacterial infection and can be activated by treatment with a needle protein (LFn-BsaL) and protective antigen (Matico et al., 2024; Yang et al., 2013). NAIP/NLRC4-dependent IL-1β release was completely blocked by VX-765 but was not inhibited by MCC950 (Fig. 2 G). BAL-0028 did not inhibit IL-1β release up to 1 μM but did reduce IL-1β at the highest dose of 10 μM (Fig. 2 G). Cell death induced by AIM2 and NAIP/NLRC4 was not affected by BAL-0028 or MCC950; at 10 μM, VX-765 also did not inhibit cell death, as higher concentrations are required to block LDH relative to IL-1β release (Schneider et al., 2017) (Fig. S2, A and B). NLRP1 is highly expressed in human keratinocytes and can be activated by the small-molecule dipeptidyl peptidase inhibitor talabostat (Val-boroPro) (Zhong et al., 2018). BAL-0028 and MCC950 have no effect on NLRP1-dependent IL-18 release, but it was completely blocked by VX-765 (Fig. 2 H). Together, these data show that BAL-0028 specifically inhibits NLRP3 but not the AIM2, NAIP/NLRC4, or NLRP1 inflammasomes at concentrations <10 μM. At high concentrations (10 μM), BAL-0028 appears to have some off-target effects that lead to partial effects on AIM2 and NAIP/NLRC4. Thus far, our data show that BAL-0028 is a specific inhibitor of NLRP3 in human myeloid cells. However, to advance BAL-0028 into *in vivo* studies in mice, we needed to determine its effects in mouse cells. Surprisingly, upon activation of NLRP3 by LPS and nigericin in the mouse macrophage cell line J774A.1, the IC_50_ of BAL-0028 for IL-1β release is increased to >6 μM (Fig. 3 A). This is a 114-fold increase over the IC_50_ for human THP-1s in the same assay (Fig. 1 B) and contrasts with MCC950, whose IC_50_ is highly consistent between the two cell lines (5.3 and 14.3 nM). We hypothesized that this difference in potency of BAL-0028 could be due to species differences in NLRP3. We therefore examined the effects of BAL-0028 and MCC950 on IL-1β release in NLRP3 activation assays (LPS + nigericin) in a range of mammalian monocytes and peripheral blood mononuclear cells (PBMCs) (Fig. 3, B–G). In cells from rat, dog, and rabbit, BAL-0028 does not block IL-1β release, whereas MCC950 potently inhibits NLRP3 activation (Fig. 3, B–D). We next examined species more closely related to humans. In cells from African green and cynomolgus monkeys (*Chlorocebus sabaeus* and *Macaca fascicularis*) BAL-0028 inhibits NLRP3-dependent IL-1β release with a potency similar to that observed for human myeloid cells (Fig. 3, E–G compared with Fig. 1, B and E–H). BAL-0028 thus appears to be a selective inhibitor of primate NLRP3 with significantly reduced potency in other species.

**Figure S3.**
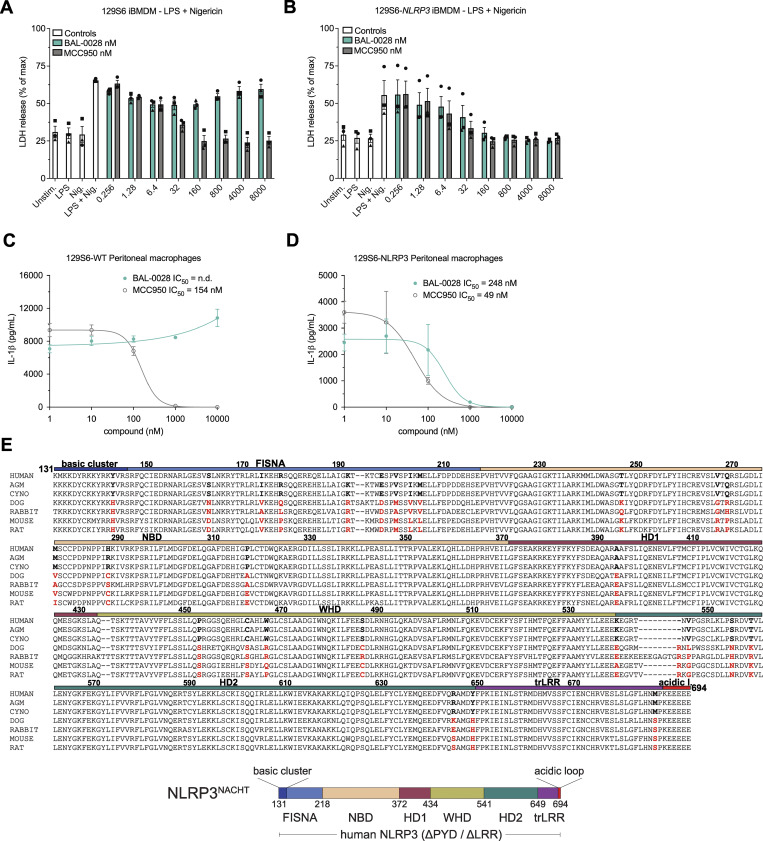
**BAL-0028 inhibits human NLRP3 in mouse cells** **.** Related to Fig. 3. **(A and B)** Comparison of BAL-0028 and MCC950 in LDH release assays from (A) WT 129S6 iBMDM and (B) 129S6-human promoter *NLRP3* iBMDM cells stimulated with LPS and nigericin. Graph symbols show average LDH values ± SEM from *N* = 3 independent experiments (indicated by different symbols) performed in triplicate. **(C and D)** Comparison of BAL-0028 and MCC950 in IL-1β release assays from (C) WT 129S6 and (D) 129S6-human promoter *NLRP3* primary peritoneal macrophages stimulated with LPS and nigericin. Graph symbols show average IL-1β values relative to vehicle control ± SD from one experiment performed in duplicate. IC_50_ curves were fitted by nonlinear regression analysis. **(E)** Multiple sequence alignment of human, African green monkey (AGM), cynomolgus monkey (CYNO), dog, rabbit, mouse, and rat NLRP3 protein sequences restricted to the corresponding sequence of amino acids 131–694 in the human NACHT domain construct shown below the alignment.

To confirm whether the difference in response to BAL-0028 observed in our human and mouse cell assays was due to species differences in NLRP3, we obtained a humanized NLRP3 mouse previously generated by Koller and colleagues where the *Nlrp3* locus is deleted and replaced with syntenic human *NLRP3* DNA, including the human promoter region (Snouwaert et al., 2016). We examined BAL-0028 and MCC950 in NLRP3 assays in immortalized bone marrow–derived macrophages (BMDM) from WT 129S6 and 129S6-*NLRP3* mice. As expected in the WT cells, BAL-0028 did not significantly inhibit IL-1β or LDH release triggered by LPS and nigericin treatment, while MCC950 potently blocked NLRP3 activation (Fig. 3 H and Fig. S3 A). However, in the *NLRP3* cells, BAL-0028 inhibits NLRP3 activation, blocking both IL-1β and LDH release with an IC_50_ of 14.7 nM for IL-1β release (Fig. 3 I and Fig. S3 B). We observed similar results in primary peritoneal macrophages, where BAL-0028 only inhibits IL-1β release in *NLRP3* cells stimulated with LPS and nigericin (Fig. S3, C and D). The potency of BAL-0028 is lower than MCC950, which is consistent with our observations in human cell assays (Fig. 1, B–E and I; and Fig. 2 B). These data indicate that the species specificity of BAL-0028 is determined by inherent differences in NLRP3 protein structure between primates and other mammals. This is a remarkable finding, as mouse and human NLRP3 are highly conserved (Anderson et al., 2004; Putnam et al., 2023). To try and identify areas that may distinguish primate NLRP3, we performed a multiple sequence alignment comparing NLRP3^NACHT^ from various species (Fig. S3 E). We focused on the NACHT domain since BAL-0028 was identified via a DEL screen using NLRP3 lacking the N-terminal PYD and was shown to interact with a construct lacking both the PYD and LRR domains in a surface plasmon resonance assay (SPR), indicating that BAL-0028 interacts with the NACHT domain (Hartman et al., 2024). While the amino acid sequences are generally conserved, there is a cluster of residues in the fish-specific NACHT associated domain (FISNA) that are distinct between humans and primates relative to mice and other mammals. Therefore, the FISNA domain may be an important determinant in how BAL-0028 interacts with NLRP3.

**Figure 4.**
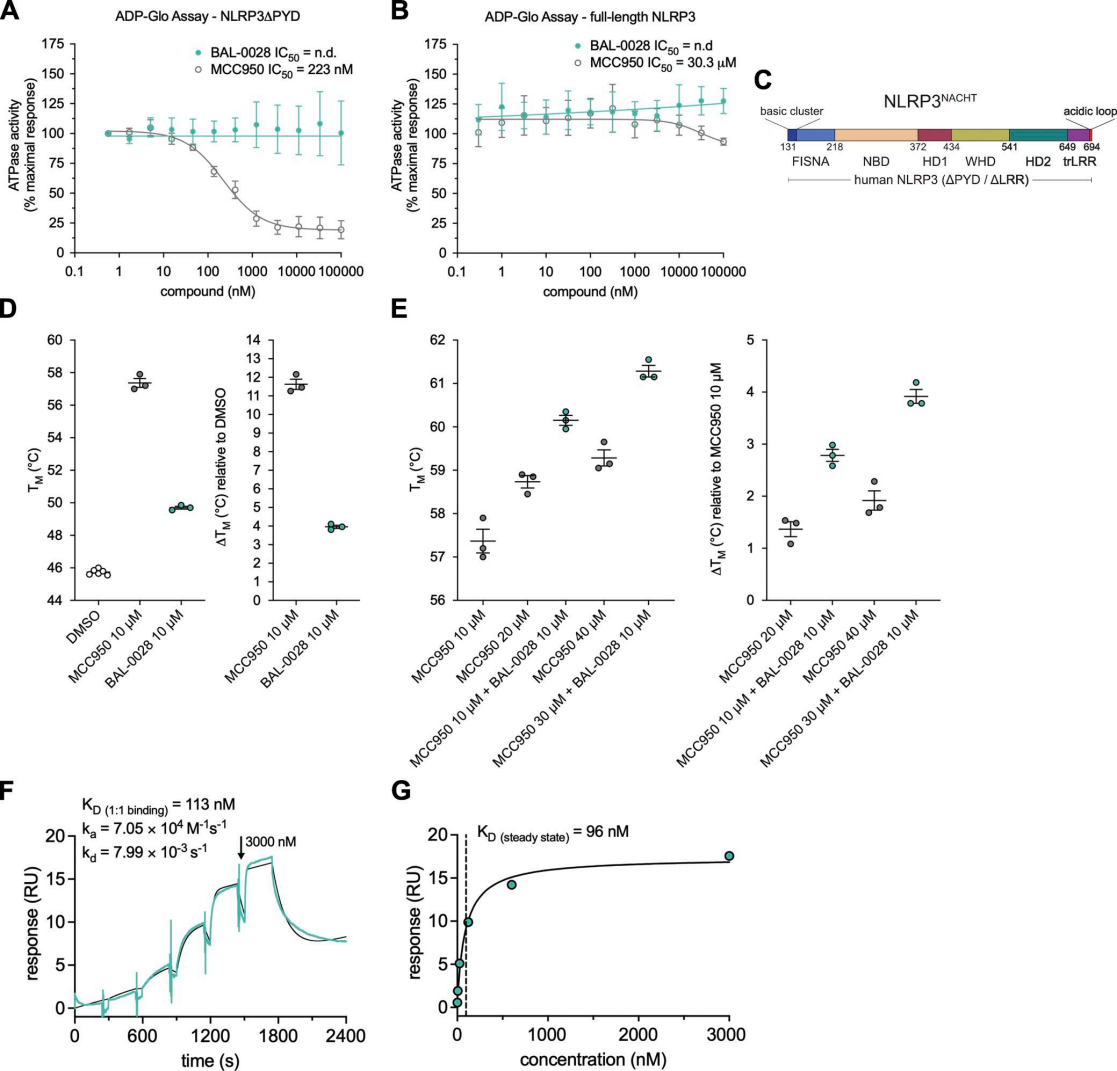
**BAL-0028 does not inhibit NLRP3 ATPase activity and binds to the NLRP3 NACHT at a site distinct from MCC950. (A and B)** Comparison of BAL-0028 and MCC950 in an ATPase activity assay with (A) recombinant MBP-ΔNLRP3-HIS protein (NLRP3ΔPYD) and (B) recombinant, full-length NLRP3 protein peak 1. Graph symbols show average values relative to vehicle control ± SEM from (A) *N* = 3 or (B) *N* = 2 independent experiments. **(C)** Schematic illustration of the NLRP3^NACHT^ recombinant protein used for nanoDSF studies. **(D and E)** nanoDSF analysis of 3 μM NLRP3^NACHT^ incubated with (D) 10 μM BAL-0028 or MCC950 or preincubated with (E) 10 μM MCC950 before addition of 10 μM BAL-0028 or 10–30 μM MCC950. Graph symbols show T_m_ or change in T_m_ relative to DMSO vehicle control or relative to MCC950-bound NLRP3^NACHT^ ± SEM from *N* = 3 independent experiments. **(F and G)** SPR analysis of BAL-0028 binding to NLRP3^NACHT^. The sensorgram of *N* = 6 injections in the single cycle measurement mode yields a kinetic *K*_D_ of 113 nM (F) and a steady-state *K*_D_ of 96 nM derived from the affinity plot (G) for the binding of BAL-0028 to NLRP3.

To examine the mechanism of action of BAL-0028, we next tested its activity in an *in vitro* ATPase assay, as many small-molecule NLRP3 inhibitors, such as MCC950, have been found to block NLRP3 ATPase activity (Coll et al., 2022). Consistent with previous reports (Yu et al., 2024), MCC950 inhibited the ATPase activity of recombinant NLRP3 lacking the PYD (NLRP3ΔPYD) (Fig. 4 A). In contrast, BAL-0028 did not have any inhibitory effect on NLRP3ΔPYD ATPase activity (Fig. 4 A). Additionally, we also performed the same ATPase assay using full-length human NLRP3, which has intrinsic ATP-hydrolysis activity (Brinkschulte et al., 2022). Full-length human NLRP3 forms a decamer where the MCC950-binding site is shielded. In agreement with Brinkschulte et al., we observed no inhibition of ATPase activity by MCC950 using this construct (Fig. 4 B). Similar to results observed with NLRP3ΔPYD, BAL-0028 did not inhibit the ATPase activity of full-length NLRP3 (Fig. 4 B). These assays confirm that the mechanism of action of BAL-0028 is distinct from MCC950 and does not involve NLRP3 ATPase inhibition.

**Figure S4.**
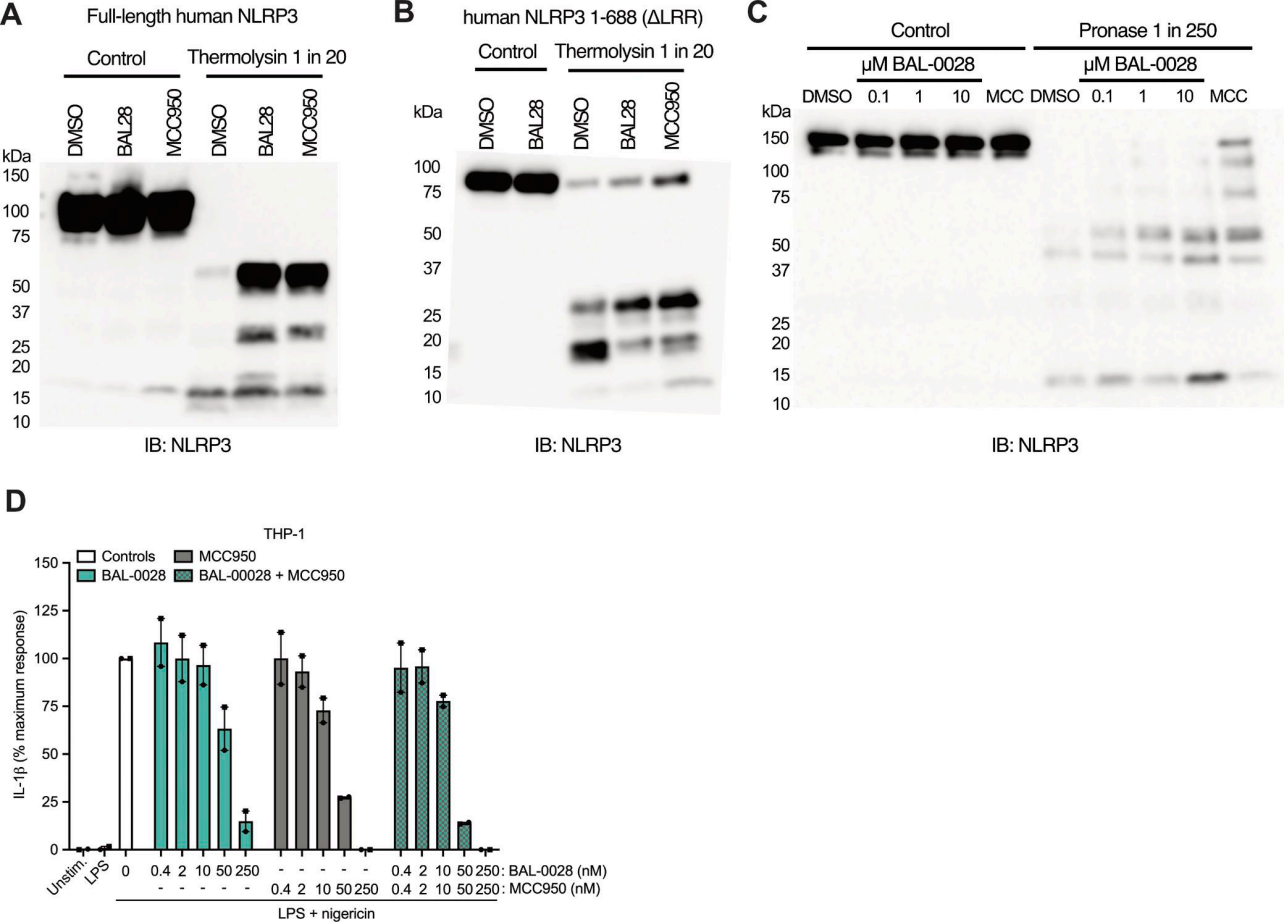
**BAL-0028 stabilizes NLRP3 in DARTS assays but does not synergize with MCC950 for NLRP3 inhibition** **.** Related to Fig. 4. **(A–C)** Western blots showing NLRP3 expression and degradation in DARTS assays performed with (A) full-length human NLRP3-Twin-Strep-tag, (B) human NLRP3 1-688-ΔLRR-Twin-Strep-tag, or (C) human-NLRP3-mCherry. Cells and cell lysates were treated with 10 μM BAL-0028 or MCC950 or DMSO control (A and B) or 0.1–10 μM BAL-0028, 10 μM MCC950, or DMSO control (C). Blots shown are representative of (A) *N* = 3, (B) *N* = 2, and (C) *N* = 4 independent experiments. **(D)** IL-1β release from PMA-differentiated THP-1 cells primed with LPS and treated with 0.4–250 nM BAL-0028 or MCC950 or both compounds together. Graph symbols show average values from independent experiments performed in triplicate (indicated by different symbols) ± SEM. *N* = 2. Source data are available for this figure: SourceData FS4.

We next examined where BAL-0028 may be interacting with NLRP3. We performed cell-based drug affinity responsive target stability (DARTS) assays with full-length human NLRP3 and NLRP3 lacking the LRR domain (NLRP3 amino acids 1–668) using BAL-0028 and MCC950. Both BAL-0028 and MCC950 prevented protease-mediated degradation of NLRP3 relative to DMSO control, as shown by the stabilization of bands in the immunoblots for NLRP3 (Fig. S4, A–C). These data support the conclusion that BAL-0028 does not interact with the LRR and instead interacts with the central NACHT region of NLRP3.

We further investigated BAL-0028’s interaction with NLRP3 using a nano-differential scanning fluorimetry (nanoDSF) approach with recombinant NLRP3 lacking the PYD and LRR domains (NLRP3^NACHT^, Fig. 4 C). Both BAL-0028 and MCC950 increase the apparent melting temperature (T_m_) of NLRP3^NACHT^, indicating that they bind to and stabilize NLRP3^NACHT^. The change in T_m_ was ∼11.5°C for MCC950 and ∼4°C for BAL-0028 (Fig. 4 D). We next performed a competition assay comparing the ability of BAL-0028 to increase the T_m_ NLRP3^NACHT^ in the presence of increasing concentrations of MCC950 (Fig. 4 E). The addition of BAL-0028 further stabilizes the protein with an increase in T_m_ of 3–4°C, whereas additional MCC950 increases the T_m_ only by 1–2°C (Fig. 4 E). Interestingly, in a THP-1 cell-based assay, BAL-0028 and MCC950 do not synergize to inhibit NLRP3 activation, and the combination of the compounds inhibits NLRP3 signaling additively (Fig. S4 D). To provide additional confirmation of a direct biophysical interaction between NLRP3^NACHT^ and BAL-0028, we performed SPR analysis (Fig. 4, F and G). BAL-0028 binds to NLRP3^NACHT^ with a steady-state K_D_ of 96 nM, the results of which are consistent with our previously published data on BAL-0028 (Hartman et al., 2024). Together, these experiments demonstrate that BAL-0028 directly binds to NLRP3^NACHT^ at a site that is distinct from the MCC950-binding pocket.

**Figure 5.**
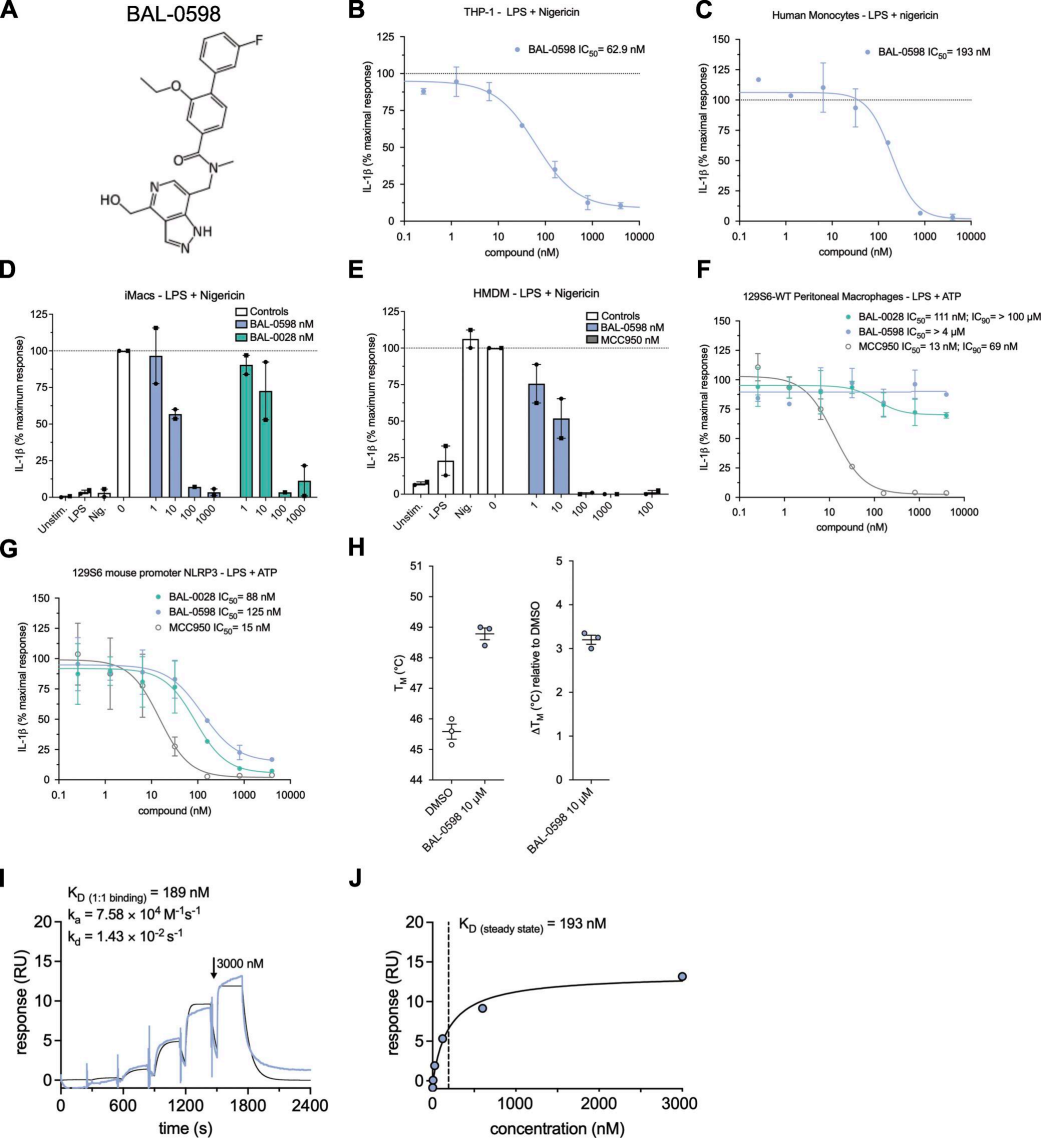
**The BAL-0028 derivative BAL-0598 inhibits human NLRP3 activation and binds directly to the NLRP3 NACHT domain. (A)** Structure of BAL-0598. **(B and C)** BAL-0598 in IL-1β release assay from PMA-differentiated THP-1 cells (B) or human monocytes (C) stimulated with LPS and nigericin. Graph symbols show average IL-1β values relative to vehicle control ± SEM from independent experiments performed in triplicate; IC_50_ curve were fitted by nonlinear regression analysis. **(D and E)** Comparison of BAL-0598, BAL-0028, and MCC950 in IL-1β release assay from (D) iMacs and (E) HMDM. Graph symbols show average values relative to vehicle control from independent experiments (indicated by different symbols) performed in triplicate ± SEM. **(F and G)** Comparison of BAL-0598, BAL-0028, and MCC950 in an IL-1β release assay in primary peritoneal macrophages isolated from WT 129S6 (F) or 129S6 mouse promoter-*NLRP3* (G) mice stimulated with LPS and ATP. Graph symbols show average IL-1β values relative to vehicle control ± SEM from independent experiments performed in duplicate. **(H)** nanoDSF measurement of 3 μM NLRP3^NACHT^ incubated with 10 μM BAL-0598. Graph symbols show T_m_ or change in T_m_ relative to DMSO vehicle control ± SEM from *N* = 3 independent experiments, each performed in duplicate. **(I and J)** SPR analysis of BAL-0598 binding to NLRP3^NACHT^. The sensorgram of *N* = 6 injections in the single cycle measurement mode yields a kinetic *K*_D_ of 189 nM (I) and a steady-state *K*_D_ of 193 nM derived from the affinity plot (J) for the binding of BAL-0598 to NLRP3. *N* = 3 (B, G, and H) and *N* = 2 (C–F).

**Figure S5.**
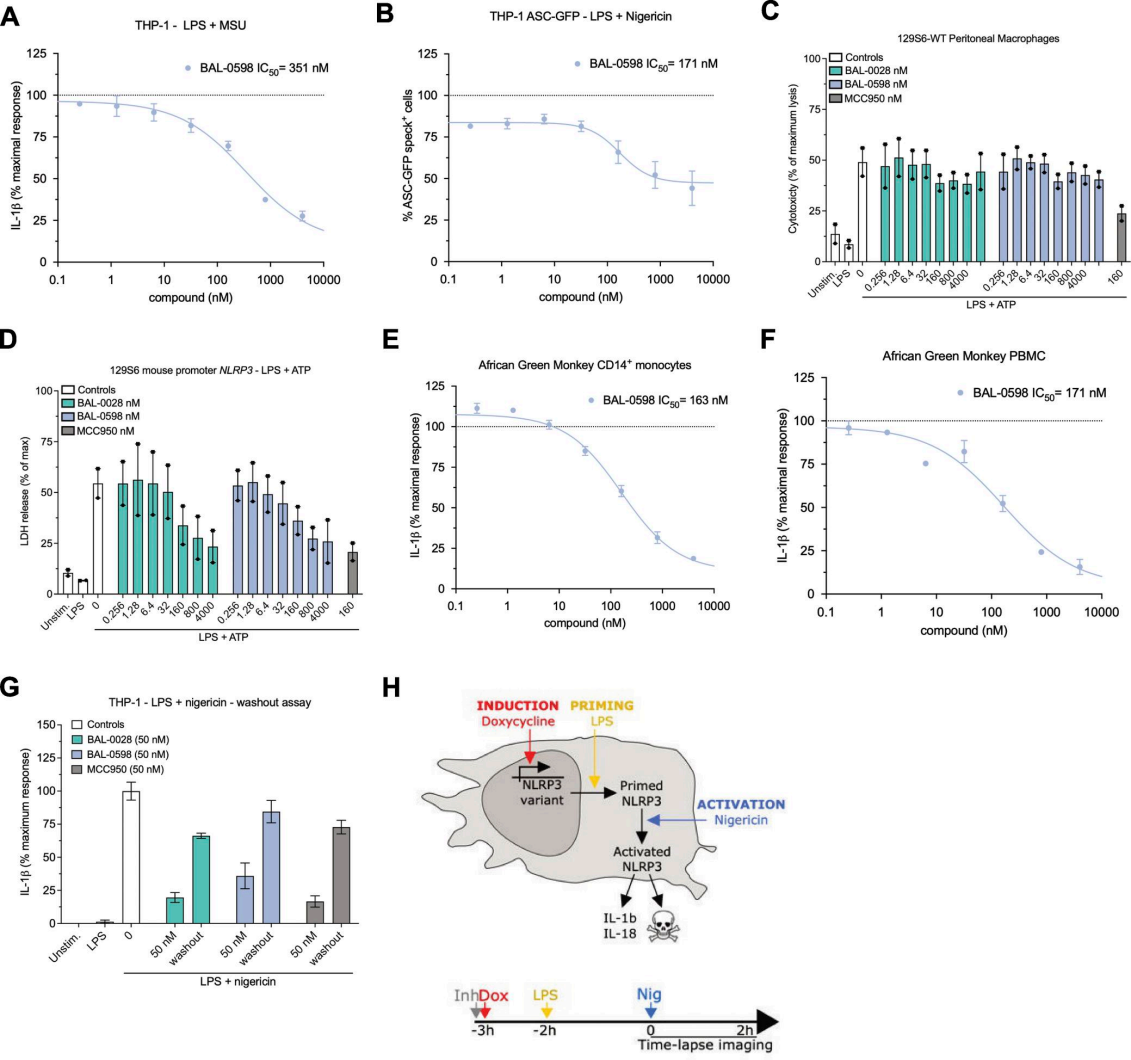
**BAL-0598 inhibits the activation of human and monkey NLRP3 and is a non-covalent inhibitor** **.** Related to Figs. 5 and 7. **(A)** BAL-0598 in IL-1β release assay from PMA-differentiated THP-1 cells stimulated with LPS and MSU. **(B)** BAL-0598 effect on ASC speck formation assessed by fluorescence microscopy in PMA-differentiated THP-1 ASC-GFP cells stimulated with LPS and nigericin. **(C and D)** Comparison of BAL-0598, BAL-0028, and MCC950 in an LDH release assay in primary peritoneal macrophages isolated from (C) WT 129S6 or (D) 129S6 mouse promoter-*NLRP3* mice stimulated with LPS and ATP. Graph symbols show average LDH values ± SEM from *N* = 2 independent experiments performed in duplicate. **(E and F)** BAL-0598 in IL-1β release assays from cells stimulated with LPS and nigericin. African green monkey CD14^+^ monocytes (E) and PBMCs (F). **(A, B, E, and F)** Graph symbols show average values relative to vehicle control ± SEM (A, *N* = 2) or SD (B, E, and F, *N* = 1) from independent experiments performed in duplicate (E) or triplicate (A, B, and F); IC_50_ curve was fitted by nonlinear regression analysis. **(G)** Comparison of BAL-0028, BAL-0598, and MCC950 in an IL-1β release assay from PMA-differentiated THP-1 cells stimulated with LPS and nigericin. Cells were treated with compounds before nigericin stimulation, and compounds were left on or were washed out for 1 min before nigericin addition. The graph shows average IL-1β values ± SD from one experiment performed in triplicate. **(H)** Schematic illustration of U937 NLRP3 and NLRP3-AID mutant cell model.

BAL-0028 has very high mouse plasma protein binding with ∼99.9% of the compound bound to plasma components (Table S1) and is therefore not optimal for use in *in vivo* efficacy studies. To address this, we generated a derivative of BAL-0028, BAL-0598, with improved pharmacokinetic properties that is ∼16 times less mouse plasma protein bound (98.39% ± 0.49%) and therefore more compatible with *in vivo* administration (Fig. 5 A and Table S1). Before embarking on *in vivo* studies, we confirmed that BAL-0598 inhibits LPS and nigericin-induced NLRP3 signaling in a range of human cell types, including THP-1, monocytes, iMacs, and HMDM (Fig. 5, B–E). BAL-0598 also inhibits NLRP3 activation by stimuli such as MSU (Fig. S5 A) and prevents NLRP3-dependent ASC speck formation (Fig. S5 B). Like BAL-0028, BAL-0598 does not inhibit NLRP3 activation in mouse macrophages (Fig. 5 F and Fig. S5 C). However, in mouse macrophages expressing human NLRP3, both BAL-0028 and BAL-0598 inhibit NLRP3 activation in a nanomolar range (Fig. 5 F and Fig. S5 D). Again, like BAL-0028, BAL-0598 inhibits primate NLRP3 as shown by NLRP3 assays in African green monkey monocytes and PBMCs (Fig. S5, E and F). These data confirm that BAL-0598 displays the same species selectivity for NLRP3 as BAL-0028. We next confirmed that BAL-0598 is a reversible inhibitor similar to BAL-0028 and MCC950 (Fig. S5 G). Finally, we confirmed that BAL-0598 directly binds to NLRP3^NACHT^ using both nanoDSF and SPR (Fig. 5, H–J). NanoDSF assays demonstrate that BAL-0598 stabilizes NLRP3^NACHT^ by 3°C, similar to our observations for BAL-0028 (Fig. 4 D). SPR showed that BAL-0598 binds NLRP3^NACHT^ with a steady-state K_D_ of 193 nM, which is similar but slightly lower than the BAL-0028 K_D_ of 98 nM (Fig. 4 G). This difference in K_D_ is reflected in the IC_50_s for cell-based NLRP3 assays with BAL-0598, which are slightly higher than those of BAL-0028 in the same conditions. For example, in THP-1 stimulated with LPS and nigericin, the IC_50_ for BAL-0598 is 62.9 vs. 57.5 nM for BAL-0028. In summary, BAL-0598 is a BAL-0028 derivative with an identical profile of NLRP3 inhibition in cell-based and biophysical assays, with a slightly reduced potency.

**Figure 6.**
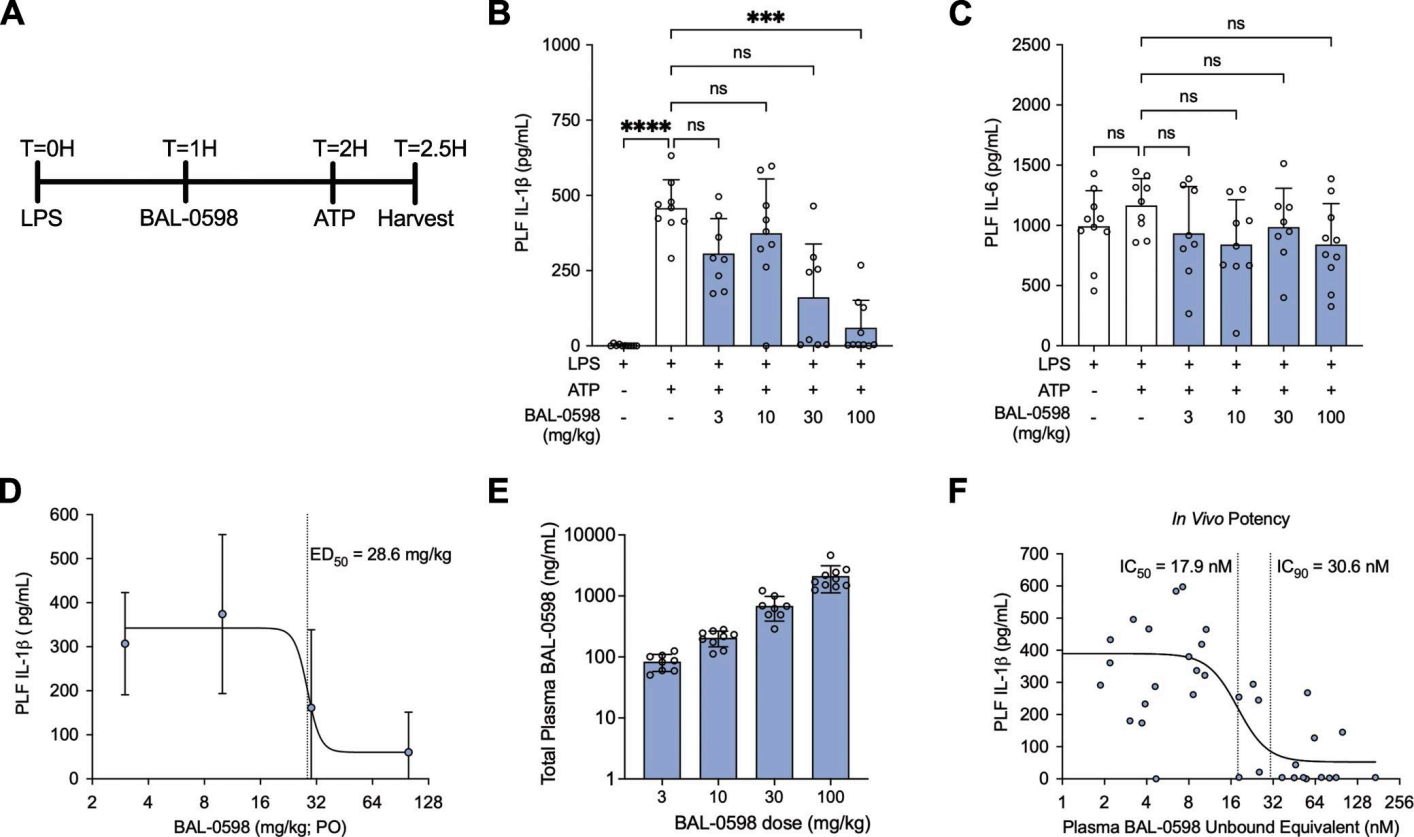
**BAL-0598 inhibits NLRP3 *in vivo*. (A)** Schematic illustration of peritonitis model and BAL-0598 dosing. **(B and C)** Levels of IL-1β (B) and IL-6 (C) in the PLF of 129S6 mouse promoter-*NLRP3* mice after peritonitis induced by LPS and ATP in the presence of increasing amounts of BAL-0598. Graph symbols show values from individual mice ± SD. **(D)** Average IL-1β release values ± SD in PLF from mice treated with BAL-0598 prior to peritonitis, and ED_50_ curve was fitted by nonlinear regression analysis. **(E)** Total plasma BAL-0598 levels from BAL-0598 oral gavage-treated mice. Graph symbols show values from individual mice ± SD. *N* = 8–10 mice per group (B–E). **(F)** IL-1β release values in PLF from 44 individual mice treated with BAL-0598 prior to peritonitis plotted against corresponding plasma levels of unbound BAL-0598, and IC_50_ and IC_90_ curves were fitted by nonlinear regression analysis. (B and C) Data were compared using a Kruskal–Wallis test with Dunn’s multiple comparisons test. ***P ≤ 0.001; ****P ≤ 0.0001.

For *in vivo* studies, we elected to use 129S6 mice where human *NLRP3* is regulated by the mouse promoter, as they have increased responses to LPS compared with human promoter *NLRP3* mice (Koller et al., 2024). To examine NLRP3 inhibition *in vivo*, we performed a well-characterized model of peritonitis using i.p. injection of LPS followed by ATP, where IL-1β production in the peritoneal cavity is NLRP3 dependent (Fig. 6 A) (Daniels et al., 2016; Pan et al., 2007; Perregaux et al., 2001). We observed a dose-dependent decrease of IL-1β in mice that received an oral dose of BAL-0598 prior to NLRP3 activation with ATP (Fig. 6 B). IL-6 levels were not attenuated by any dose of BAL-0598 (Fig. 6 C). The effective dose (ED_50_) of BAL-0598 to reduce the IL-1β response by half is ∼28.6 mg/kg (Fig. 6 D).

To better understand how systemic exposure of BAL-0598 correlates to IL-1β release in the peritoneal cavity, *in vivo* potency was determined by plotting the unbound plasma levels of BAL-0598 against the absolute IL-1β concentrations measured in the peritoneal lavage fluid (PLF) of individual mice. Just prior to lavage of the peritoneal cavity, terminal plasma samples were collected, and the total plasma concentrations of BAL-0598 were determined by liquid chromatography–tandem mass spectrometry (LC-MS/MS) (Fig. 6 E). The unbound BAL-0598 equivalent for each mouse was then calculated using the mean unbound protein plasma-binding value of 1.61% (Table S1). The *in vivo* dose–response inhibition curve indicates that after oral dosing, the unbound systemic exposure of BAL-0598 required to reduce the IL-1β response by half is ∼17.9 nM, and by 90% is 30.6 nM (Fig. 6 F). Importantly, the *in vivo* potency data indicate that the ability of BAL-0598 to inhibit peritoneal macrophages activated with LPS and ATP *ex vivo* underestimates the inhibitory activity of BAL-0598 *in vivo* (Fig. 5 G).

**Figure 7.**
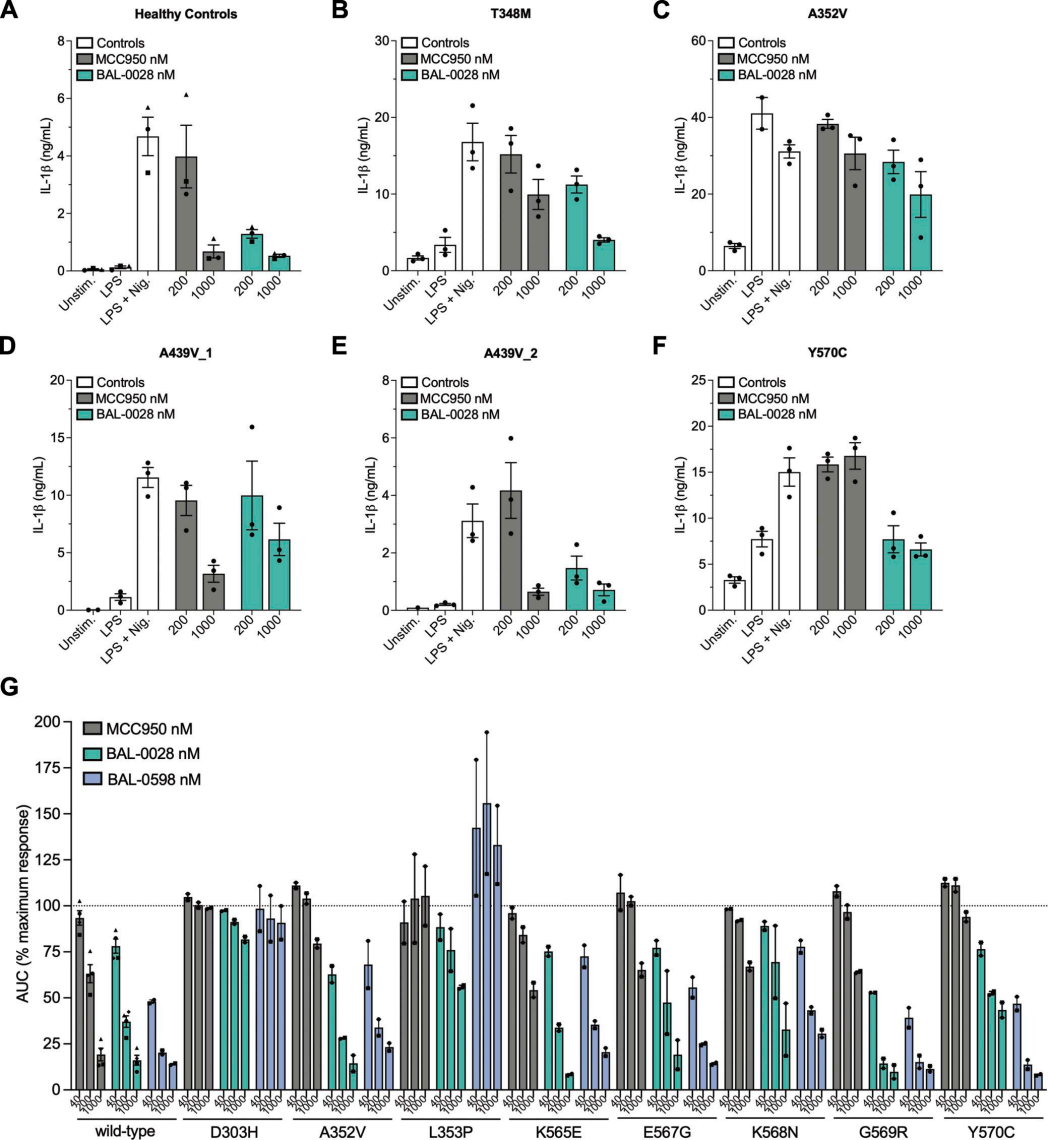
**BAL-0028 and BAL-0598 inhibit NLRP3-AID mutants. (A–F)** IL-1β release assays from LPS and nigericin-stimulated PBMCs pre-treated with MCC950 (200, 1,000 nM) or BAL-0028 (200, 1,000 nM). Data from (A) healthy controls or patients with NLRP3-AID mutations (B) T348M, (C) A352V, (D and E) A439V, and (F) Y570C. Graph symbols show average IL-1β values ± SEM from *N* = 3 (A) or *N* = 1 (B–F) independent experiments performed in biological triplicate. **(G)** Comparison of BAL-0028 and MCC950 (40–1,000 nM) in a cell death assay in U937 cells expressing NLRP3 or NLRP3-AID mutants (D303H, A352V, L353P, K565E, E567G, K568N, G569R, and Y570C) stimulated with LPS and nigericin. Graph symbols show average area under the curve (AUC) values relative to DMSO vehicle control for each cell type ± SEM from *N* = 2–4 independent experiments performed in duplicate.

To examine if BAL-0028 could inhibit disease-associated NLRP3 mutations, we first tested primary human PBMCs from five NLRP3-AID patients and healthy controls. As expected, both MCC950 and BAL-0028 effectively blocked IL-1β release from healthy control PBMCs stimulated with LPS and nigericin (Fig. 7 A). BAL-0028 also inhibited IL-1β release from patient PBMCs with T348M, A352V, A439V (two patients), and Y570C NLRP3 mutations (Fig. 7, B–F). Interestingly, BAL-0028 inhibited more effectively than MCC950, particularly in the constitutively active mutations A352V and Y570C (Fig. 7, C and F).

Finally, to more thoroughly investigate whether BAL-0028 or BAL-0598 could be used to treat disease-associated NLRP3 mutations, we employed a U937 cell model to express gain-of-function NLRP3-AID mutants as previously described (Cosson et al., 2024) (Fig. S5 H). NLRP3 or NLRP3-AID mutant expression is induced by doxycycline treatment in U937s, and NLRP3 is activated by stimulation with LPS and nigericin. We selected eight NLRP3-AID gain-of-function mutations, including known mutations shown to be relatively insensitive to MCC950 inhibition (D303H, L353P, and G569R) and well-characterized constitutively active mutants (A352V, K565E, E567G, K568N, and Y570C) (Cosson et al., 2024). Pre-treatment of WT NLRP3 with MCC950, BAL-0028 or BAL-0598 (40–1,000 nM) dose dependently inhibits cell death (Fig. 7 G). However, in NLRP3 mutant cells, MCC950 fails to inhibit (D303H and L353P) or partially inhibits cell death at 1,000 nM (A352V, K565E, E567G, K568N, G569R, and Y570C), as expected. In contrast, BAL-0028 and BAL-0598 potently inhibit A352V, K565E, E567G, K568N, and G569R at levels similar to WT NLRP3. Interestingly, while BAL-0028 partially inhibited D303H and L353P at 1,000 nM, BAL-0598 did not inhibit these mutations. However, BAL-0598 more potently inhibited Y570C than BAL-0028. In addition, the inhibitory activity of BAL-0028 in the U937 model is consistent with our primary PBMC data for A352V and Y570C. Together, these data demonstrate the ability of BAL-0028 and BAL-0598 to inhibit disease-associated NLRP3 mutations, and particularly those that may be insensitive to MCC950-like small molecules.

## Discussion

Previous efforts to identify NLRP3 inhibitors have largely focused on cell-based phenotypic screens in mouse and human myeloid cells. As NLRP3 activation is a highly complex process involving multiple priming and activation mechanisms for different stimuli (McKee and Coll, 2020; Swanson et al., 2019), deconvolving “hits” from such cellular screens can be extremely challenging. We therefore chose an alternative approach and employed a DEL screen to identify compounds that interact with recombinant NLRP3, yielding the lead compound BAL-0028 (Hartman et al., 2024). Here we have characterized the mode of action of BAL-0028 in depth, benchmarking it with the tool molecule MCC950. We observe that BAL-0028 inhibits NLRP3 signaling in a range of human cell types and in response to multiple NLRP3 stimuli (Figs. 1 and 2). BAL-0028 is a specific inhibitor of NLRP3, as it does not prevent activation of the NLRP1, NLRC4, or AIM2 inflammasomes and does not affect LPS-induced signaling (Figs. S1 and 2). In these human cell–based NLRP3 activation assays, BAL-0028 effectively phenocopies MCC950, albeit at slightly higher concentrations.

Surprisingly, when we examined BAL-0028 in mouse macrophages, we noticed a highly significant decrease in its ability to inhibit NLRP3 (Fig. 3). A recent study also described an NLRP3 inhibitor, NDT-0796, that is active in human cells but inactive in mouse macrophages (Smolak et al., 2024). This difference was because NDT-0796 is a prodrug that must be metabolized by carboxylesterase-1 (CES-1) into its active form, and mouse macrophages do not express CES-1 (Smolak et al., 2024). As BAL-0028 does not contain a carboxylate, this explanation for the species differences observed was unlikely. A molecule termed J114 that inhibits both NLRP3 and AIM2 inflammasomes is also reported to be significantly less potent in mouse macrophages relative to human cells (Jiao et al., 2022). However, the reason for this species difference was not examined. We chose to explore the ability of BAL-0028 to inhibit NLRP3 in other species, and while BAL-0028 could not inhibit rat, dog, or rabbit NLRP3, it potently blocked monkey NLRP3 (Fig. 3). Similarly, BAL-0598 inhibits human and monkey NLRP3 but not mouse NLRP3 (Figs. 5 and S5). BAL-0028 and BAL-0598 are thus active in humans and closely related monkey species, making them the first primate-specific NLRP3 inhibitors to be reported. This species specificity is surprising given the high sequence conservation of the NACHT domain in mammalian NLRP3 (Anderson et al., 2004), but it is consistent with the use of human NLRP3 protein in the DEL-screening assay. In line with this, our studies using 129S6 mice expressing human NLRP3 unequivocally show that the potency of BAL-0028 and BAL-0598 is dependent on the differences between mouse and human NLRP3 (Figs. 3, 5 and S5). Thus, even subtle changes in NLRP3 structure between mouse and human can be exploited for drug development. While much of our knowledge of inflammasome biology has been informed by mouse models, there are many differences in inflammasome structure, expression, and regulation in humans. For example, NLRP1 biology has significantly advanced in recent years due to studies on keratinocytes, which express NLRP1 in humans but not in mice. NLRP1 is also structurally distinct in mouse, where it lacks a PYD (Barry et al., 2023). There are also two families of regulatory proteins termed pyrin-only proteins (POPs) and CARD-only proteins (COPs) that only arose in the primate lineage. COPs and POPs are known to regulate inflammasome formation and signaling, but functional studies have been relatively limited as they are not expressed in mice (Devi et al., 2020). Our work highlights the importance of performing screens using human proteins and cell systems to capture these important biological differences in the immune system.

The species-specific effects of BAL-0028 and BAL-0598 also reveal a clear difference with MCC950, which inhibited NLRP3 in all species tested. Our mechanistic studies further show that BAL-0028 does not inhibit the ATPase activity of NLRP3, distinguishing it from MCC950 and many other NLRP3 inhibitors, such as CY-09, that appear to converge on inhibiting NLRP3 via blocking this ATPase activity (Coll et al., 2022). We measured the ATPase activity of both NLRP3ΔPYD and full-length NLRP3 and confirmed the results of Brinkschulte et al. (2022), which observed that MCC950 could not inhibit full-length NLRP3 ATPase activity. We believe this difference can be explained by the different protein constructs used, which lead to different quaternary assemblies of the NLRP3 protein and different accessibility of the MCC950-binding site. Whereas full-length, WT human NLRP3 forms a decamer where the MCC950-binding site is shielded in the inactive conformation and the nucleotide-binding domain occupied with ADP, a construct lacking the N-terminal PYD was shown to form a hexamer through the back-to-back LRR assembly, leaving the MCC950- and nucleotide-binding sites accessible (Ohto et al., 2022). We conclusively show that BAL-0028 and BAL-0598 bind to NLRP3 in the NACHT domain using DARTS, nanoDSF, and SPR assays. Indeed, our nanoDSF analysis reveals a novel synergistic stabilization of NLRP3^NACHT^ by BAL-0028 in the presence of MCC950, showing that they bind at different sites of the protein. BAL-0028 therefore inhibits NLRP3 by a mechanism distinct from MCC950 and many other previously characterized NLRP3 inhibitors. Based on our analysis of NLRP3 sequences, we speculate that BAL-0028 may interact in the FISNA subdomain of the NLRP3^NACHT^, although it is not currently understood how this proposed binding interaction is consistent with the computer-aided drug design–binding site prediction previously published for BAL-0028 (Hartman et al., 2024). While we have not experimentally identified the specific binding site of BAL-0028 and BAL-0598, this is the subject of ongoing structural biology studies.

For the future development of BAL-0028–related compounds, establishing *in vivo* efficacy is important. As a first step, we demonstrated that BAL-0028 is active in *ex vivo* human whole blood NLRP3 assays (Fig. 1); however, we developed an analog, BAL-0598, for *in vivo* studies due to the high plasma protein binding of BAL-0028. In the human NLRP3 129S6 mice in a peritonitis model, we show that BAL-0598 dose dependently blocks IL-1β release but not IL-6 (Fig. 6). Furthermore, our *in vivo* peritonitis studies indicate that *ex vivo* cell-based potency assays performed in peritoneal macrophages underestimated the *in vivo* potency after oral dosing of BAL-0598, based on the *in vivo* dose–response inhibition curve derived from unbound BAL-0598 plasma values and absolute IL-1β in the PLF.

Using both primary human NLRP3-AID PBMCs and a cell-based model of NLRP3-AID, we show that BAL-0028 and BAL-0598 can also inhibit gain-of-function NLRP3 mutations. All the mutations we examined are located in the NACHT within the nucleotide-binding domain or helical domain 2, causing constitutively active or hyperactive NLRP3 variants. A number of these mutations (D303H, K568N, and Y570C) are known to cause the severe NLRP3-AID neonatal-onset multisystem inflammatory disease (Infevers Database) and include several somatic mutations (D303H, K565E, E567G, K568N, and Y570C) (Cosson et al., 2024). BAL-0028 and BAL-0598 were able to fully or partially inhibit these highly pathogenic NLRP3 mutations, suggesting that BAL-0028 derivatives can be effective treatments for many NLRP3-AID disease variants. Importantly, for many of these mutations, BAL-0028 and BAL-0598 were more effective inhibitors than MCC950 (Fig. 7). BAL-0028 or its derivatives like BAL-0598 may thus be an alternative therapeutic modality for NLRP3-AID patients that may not respond to MCC950-derived molecules.

In summary, we have characterized novel small-molecule inhibitors that are potent and specific for primate NLRP3. We anticipate that future work on BAL-0028 and BAL-0598 will reveal novel insights into the biology of NLRP3 and that improved understanding of the structural biology of human NLRP3 will inform the development of BAL-0028–related compounds.

## Materials and methods

### Source of BAL-0028, BAL-0598, and MCC950

The synthesis of BAL-0028 and BAL-0598 is described in detail in US patents 11708334 and 11708334 (Hartman et al., 2023a; Hartman et al., 2023b). MCC950 was purchased from Adipogen, ApexBio, or Selleck Chemicals.

### THP-1 cell–based assays

Human monocytic THP-1 cells (ATCC) were cultured in growth media (THP GM) until they reached logarithmic growth and achieved a viability >90%. THP GM is composed of RPMI-1640 + GlutaMAX (Gibco)/10% FBS (Corning)/55 µM β-mercaptoethanol (Gibco)/penicillin-streptomycin (pen/strep) (Caisson). Cells were spun down and resuspended to 1,000,000 cells/ml in THP GM containing either 20 or 500 nM PMA (Sigma-Aldrich). 150,000 cells (150 μl) were then added to each well of a 96-well TC plate and incubated for either 24 h or 3 h, respectively, in a standard cell culture incubator (37°C; 5% CO_2_). In the NLRP3 inflammasome activation assays, 200 μl of THP GM or THP GM containing 100 ng/ml LPS (*Escherichia coli* O26:B6; Sigma-Aldrich) was then added to the appropriate wells, and the cells were incubated for an additional 3 h. The media was then replaced with Opti-Mem medium (without serum; Thermo Fisher Scientific) containing predetermined dilutions of test compounds in replicate wells. After a 30-min incubation, 10 µM nigericin (final concentration; Sigma-Aldrich) in Opti-Mem medium with the corresponding concentration of test compound was added to the wells for an additional 1 h. Positive control wells contain 10 µM nigericin in Opti-Mem in the absence of test compound, while negative control wells contain Opti-Mem only. Supernatants were then assayed for IL-1β (human; DuoSet; R&D) and relative LDH levels (as a surrogate for pyroptosis) using a CytoTox 96 Kit (Promega). For the THP-1 LPS signaling assays, 200 μl of THP GM (negative control wells), THP GM containing 100 ng/ml LPS (positive control wells), or THP GM containing 100 ng/ml LPS with predetermined dilutions of test compounds in replicate wells was added to the appropriate wells for 3 h. Supernatants were then assayed for IL-6 (human; DuoSet; R&D), TNF (human; DuoSet; R&D). and relative LDH levels using a CytoTox 96 Kit (Promega). For THP-1 viability assays, after the PMA incubation step, the media was replaced with Opti-Mem medium (without serum; Thermo Fisher Scientific) or Opti-Mem medium containing predetermined dilutions of test compounds in replicate wells and incubated for 1.5 h. Supernatants were then assayed for relative LDH levels using a CytoTox 96 Kit (Promega). In all THP-1 assays, once supernatants were removed, the relative viability of adherent cells in the 96-well TC plate was determined using a CellTiter-Glo luminescent cell viability assay (Promega).

### Human microglial NLRP3 inflammasome activation assay

Human iCell microglia (R1131; Fujifilm) were directly thawed in poly-L-lysine–coated 96-well plates at ∼50,000 cells/well in iCell glial base medium supplemented with iCell microglia supplements A, B, and C as per the manufacturer’s instructions. Cells were incubated at 37°C and 5% CO_2_. Next day, 50% of the glial medium was replaced with fresh medium, and cells were incubated for a further 2 days. For potency determination, iCell microglia were first primed with 200 ng/ml LPS for 4 h and then preincubated for 30 min with fivefold compound concentrations ranging from 0.256 nM to 4 µM. Cells were then stimulated with 10 µM nigericin with the corresponding compound concentrations and incubated for an additional 30 min. Supernatants were collected for cytotoxicity assay and cytokine ELISAs.

### HMDM NLRP3 inflammasome activation assay

Buffy coats were obtained from the Northern Ireland Blood Transfusion Service (reference number 2019/05), and ethical approval for the use of buffy coats was obtained from the Queen’s University Faculty Research Ethics Committee (reference MHLS 19_17). PMBCs were isolated by density centrifugation using Ficoll-Paque Plus (12440053; GE Healthcare). CD14^+^ monocytes were then isolated using magnetic-activated cell sorting CD14-positive selection (130-050-201; Miltenyi Biotech) according to the manufacturer’s instructions. CD14^+^ monocytes were cultured in RPMI-1640, 10% FCS, 50 U/ml pen/strep (all Gibco), and 50 ng/ml recombinant human macrophage CSF (M-CSF) (11343118; ImmunoTools) for 7 days. HMDM were seeded at 0.7 × 10^6^ cells/ml in 96-well TC plates. For NLRP3 assays, the following day the media was removed and replaced with Opti-Mem ±100 ng/ml Ultrapure LPS from *E. coli* K12 (InvivoGen) for 4 h. Test compounds were prepared by serial dilution in DMSO. The media were removed and replaced with Opti-Mem containing inhibitors or 0.1% DMSO vehicle control. After 30-min incubation, 5 μM nigericin (Adipogen) was added for 2 h. Cell-free supernatants were removed, and IL-1β and TNF were assessed by ELISA according to the manufacturer’s instructions (88-7261-77, 88-7346-77; Thermo Fisher Scientific).

### Human iMacs NLRP3 inflammasome activation assay

KOLF2-C1 iPSCs were obtained from the Wellcome Sanger Institute (Hinxton, UK). iPSCs were cultured on vitronectin-coated (A14700; Thermo Fisher Scientific) plates in Essential 8 medium (A1517001; Thermo Fisher Scientific) at 37°C in a humidified 5% CO_2_ incubator with 10 μM Rho-kinase (Rock) inhibitor Y-27632 dihydrochloride (Y0503; Merck) added following thawing and passaging. Embryoid bodies (EBs) were formed as previously described (Douthwaite et al., 2022). In brief, iPSCs were seeded at 1 × 10^5^ cells in 100 μl per well onto low-adherent, U-bottom 96-well plates in Essential 8 medium supplemented with 50 ng/ml BMP-4 (314-BP010; R&D Systems) and 10 μM Rock inhibitor. The plates were centrifuged for 3 min at 800 × *g*, 4°C, and incubated for 5 days. The EBs were then transferred to gelatine-coated (G1890; Sigma-Aldrich) tissue culture flasks in myeloid precursor base medium: X-VIVO 15 Serum Free Medium (BE02-060F; Lonza), 1× GlutaMAX, and 1% pen/strep. Media was changed every 4–5 days, and the EBs began to produce myeloid precursor cells approximately 3–4 wk after transferring to the gelatine-coated flasks. To harvest the precursor cells, the medium was collected from the EB flasks and centrifuged for 3 min at 300 × *g*, and the cells were resuspended in iMac medium: RPMI-1640, 10% FBS, 1× GlutaMAX, and 1% pen/strep supplemented with 100 ng/ml M-CSF (HZ-1192; Proteintech). Myeloid precursor cells were differentiated for 7 days and then harvested using lidocaine monohydrate (L5647; Sigma-Aldrich) and plated onto 96-well TC plates at 0.5 × 10^6^/ml in 100 μl per well in iMac medium. NLRP3 assays were conducted as described for HMDM above, except cytotoxicity was also assessed in cell-free supernatants by LDH assay (MK401; Takara Bio).

### Human whole blood assays

The human whole assays followed approximately the procedure described previously (Grinstein et al., 2018). A human blood sample was collected by a licensed phlebotomist in sodium heparin-containing tubes in accordance with Protocol #20223689 approved by the WCG Institutional Review Board (IRB00000533). 140 μl of heparinized whole blood was then added to each well of a 96-well TC plate. 20 μl of PBS (no Ca^2+^ and no Mg^2+^), PBS containing 8 µg/ml LPS (*E. coli* O26:B6; Sigma-Aldrich; final concentration 1 µg/ml), or PBS containing 8 µg/ml LPS (final concentration 1 µg/ml) with predetermined dilutions of test compounds in replicate wells was added to the wells for 3 h on a shaker (180 rpm) in a standard cell culture incubator (37°C; 5% CO_2_). After 3 h, 40 μl of 5 mM ATP (A6419; Sigma-Aldrich; final concentration 1 mM) was added to the appropriate wells and incubated for an additional 30 min on a shaker (180 rpm). At the end of all treatments, 100 μl of PBS was added to each well, the plates were centrifuged at 450 × *g* for 15 min at RT, and the supernatant from each well was assayed for IL-1β (human; DuoSet; R&D) or IL-18 (human; DuoSet; R&D).

### Western blots

THP-1 cells are cultured as described above with the following differences: 4.5 × 10^6^ cells were seeded in THP GM containing 500 nM PMA (Sigma-Aldrich) into each well of a 6-well cell culture plate and incubated for 3 h. After this incubation, the plate was tilted and media carefully removed. 2 ml of THP GM or THP GM containing 100 ng/ml LPS (*E. coli* O26:B6; Sigma-Aldrich) is then added to the appropriate wells, and the cells are incubated for an additional 3 h. The media is again removed and replaced with Opti-Mem medium (without serum; Thermo Fisher Scientific) OR Opti-Mem medium (without serum; Thermo Fisher Scientific) containing 200–500 nM of test compound. After a 30-min incubation, 10 µM nigericin (final concentration; Sigma-Aldrich) in Opti-Mem medium with 200–500 nM of test compound is added to the wells for an additional 30 min. Positive control wells contain 10 µM nigericin in Opti-Mem in the absence of test compound, while negative control wells (PMA or PMA- + LPS-treated cells) contain Opti-Mem only. Supernatants are collected and condensed 10-fold by centrifugation using Pierce Protein Concentrator columns (88512; Thermo Fisher Scientific) according to the manufacturer’s supplied instructions. NuPAGE LDS sample buffer (NP0007; Thermo Fisher Scientific) with reducing agent (NP0009; Thermo Fisher Scientific) is then added to the appropriate volume of condensed supernatant samples for gel loading. The adherent cells were then washed two times with ice-cold TBS; all liquid was subsequently removed, and the cells were lysed with 100 μl per well of 1× radioimmunoprecipitation assay (RIPA) buffer (RB4475; BioBasic) containing protease cocktail (P8340; Sigma-Aldrich) and caspase-1/4 inhibitor VX-765 (inh-vx765i-1; InvivoGen) while on ice. Insoluble cellular material was removed by cold centrifugation at 16,000 × *g*. The lysates were then resuspended with 1× RIPA buffer and sample buffer with reducing agent to a final concentration of 0.4 mg/ml. 10 µg of protein was loaded per well of a 10% Tris-Bis gel (NP0301BOX; Thermo Fisher Scientific) and run at 100 V for approximately 2–3 h, after which the proteins were transferred to a PDVF membrane using an Iblot (IB1001) transfer apparatus at 20 V for 7 min. Membranes were then blocked in TBS-T (10 mM Tris-HCl (pH 7.4), 150 mM NaCl, 0.2% Tween-20) containing 5% BSA (AAJ65097-22; VWR) at RT for 45 min while shaking. Membranes were then incubated with one of the following primary antibodies: NLRP3 (15101; Cell Signaling), cleaved IL-1β (12242; Cell Signaling), cleaved caspase-1 (4199; Cell Signaling), and actin (A2066; Sigma-Aldrich). After incubation, the membranes were washed and incubated with one of the compatible secondary antibodies: anti-mouse (115-035-062; Jackson) or anti-rabbit (111-035-144; Jackson) in TBS-T. Proteins were detected by incubating the membrane with Femto SuperSignal substrate (34095; Thermo Fisher Scientific), and a luminescence signal was detected using a BioRad ChemiDoc XRS+ Imaging System.

### ASC speck assays

THP-1-ASC-GFP (InvivoGen) were maintained in growth media containing RPMI-1640 + GlutaMAX (Gibco) with 10% FBS (Corning) and 1% pen/strep (Caisson) at 37°C in a 5% CO_2_ atmosphere. Cells were seeded in a 96-well plate (150,000 cells/well) in growth media containing 100 nM PMA for 3 h. PMA-treated cells were primed with 100 ng/ml LPS (O6:B26; Sigma-Aldrich) for a further 3 h, followed by a 30-min preincubation with test compounds. To activate NLRP3, cells were stimulated with 10 µM nigericin with and without test compounds for 30 min. Nuclei were stained with the live stain, Hoechst 33342, for 5 min. Cells were imaged live (20× magnification) and analyzed using the CellInsight CX5 high-content screening platform (Thermo Fisher Scientific ). Each well was imaged in two channels (GFP and DAPI) for nine fields per well. The number of nuclei (DAPI) and perinuclear ASC specks (GFP) were counted, and the percent of ASC specks/nuclei was calculated. For IC_50_ determination, the % ASC speck-positive cells were plotted against BAL-0028 or MCC950 concentrations (nM).

### HEK293T cell ASC speck formation assay

HEK293T cells stably expressing ASC-BFP fusion protein were used as previously described (Hochheiser et al., 2022a). Cells were seeded into 24-well TC plates at a density of 125,000 cells per well and incubated overnight at 37°C. To induce moderate expression of NLRP3 for assessable ASC speck formation, cells were transfected with 100 ng per well of a doxycycline-inducible TetO6-NLRP3-hPGK-TetON3G-T2A-mCherry construct using Lipofectamine 2000 (Thermo Fisher Scientific) according to the manufacturer’s instructions. 16 h after transfection, NLRP3 expression was induced by adding doxycycline (10 ng/ml). Simultaneously, cells were treated with increasing concentrations of BAL-0028 or MCC950 and incubated for 4 h. To induce ASC speck formation, cells were subsequently treated with 10 µM nigericin for 1 h. After trypsinization, cells were washed and resuspended in flow buffer (DPBS, 2 mM EDTA, and 0.5% BSA). Flow cytometry was conducted at an LSRFortessa II. Gates were set to select for single cells expressing ASC-BFP. From these, mCherry-positive, NLRP3-expressing cells were selected. Among the mCherry-positive cells, the proportion of ASC speck formation was calculated as previously described by Sester et al. (2015). The difference in ASC speck formation between nigericin-stimulated and unstimulated cells was plotted against increasing antagonist concentrations. These values were normalized to the speck formation difference calculated for cells without compound treatment (% maximum response). The generated dose–response curve was fitted using the four-parameter logistic equation built into Prism 10.1.1. to determine the half-maximal inhibitory concentrations of BAL-0028 and MCC950.

### iMacs ASC speck staining and imaging

Cells were plated at 1 × 10^5^ per well overnight in 24-well plates with coverslips in 500 μl iMac medium. LPS (100 ng/ml) was used to prime the cells for 3.5 h, and MCC950 (1 μM) or BAL-0028 (1 μM, 100 nM, or 10 nM) was added for 30 min prior to the addition of nigericin (5 μM) for 2 h. Cells were washed in PBS, fixed in 4% paraformaldehyde for 15 min, and stored in PBS until staining. Coverslips were washed 3 times in PBS, then permeabilized with 0.1% Triton X-100 in PBS for 10 min. Coverslips were washed three times in PBS, then 50 mM ammonium chloride was added for 10 min to quench the paraformaldehyde. Coverslips were washed three times in PBS and then blocked with 0.5% BSA/PBS for 30 min. Primary anti-ASC antibody (676502; BioLegend) was diluted 1 in 50 in 0.5% BSA/PBS and added for 60 min, then coverslips were washed in PBS four times. Alexa Fluor 488 Donkey anti-mouse (A21202; Invitrogen) was diluted 1 in 800 in 0.5% BSA/PBS and added for 1 h. The coverslips were washed four times in PBS before mounting in Vectashield Antifade Mounting Medium with DAPI (H-1299; 2BScientific) on glass microscope slides. The mounted coverslips were sealed with clear nail polish and stored at 4°C until imaging. Coverslips were imaged using the Leica LAS X software and the DM5500 fluorescent microscope at 20× and 40×. Six fields of view were analyzed per condition in each experiment with two to three technical replicates (coverslips) for each biological replicate. ASC specks were quantified using Fiji. An automated system was used to quantify the number of DAPI^+^ cells, and the ASC specks were manually counted. The percentage of DAPI^+^ASC^+^ positive cells was calculated.

### AIM2, NAIP/NLRC4, and NLRP1 inflammasome activation assays

Monocytic THP-1 cells were seeded in 96-well plates (150,000 cells/well) in growth media containing 100 nM PMA for 3 h. For NAIP/NLRC4, growth media was aspirated, and 180 μl of Opti-MEM with 10-fold BAL-0028 or MCC950 (Selleck Chemicals) concentrations ranging from 10 nM to 10 µM was added to the cells. Then, 20 μl of 10× “NeedleTox” comprising a mixture of 5 µg/ml LfN-Needle (InvivoGen) and 5 µg/ml anthrax toxin’s protective antigen (EMD Millipore) was added to activate NAIP/NLRC4 (final concentration 5 ng/ml). Cells treated with NeedleTox (positive control) and cells with OptiMEM alone (negative control) were included in the experiment. For AIM2, PMA-treated THP-1 cells were primed with 100 ng/ml LPS (O6:B26; Sigma-Aldrich) for 24 h. Cells were then incubated with the AIM2 ligand, polydA:dT/Lyovec (InvivoGen; final concentration 5 µg/ml), with and without BAL-0028 or MCC950, 10-fold concentrations ranging from 10 nM to 10 µM. For NAIP/NLRC4 and AIM2 inflammasomes, cells were incubated at 37°C and 5% CO_2_ for 24 h, and supernatants were collected for cytokine analysis. The caspase-1 inhibitor, VX-765 (InvivoGen; final concentration 10 µM), was used as a positive control for inflammasome inhibition. For NLRP1, human keratinocytes (NHEK; Lonza) were used for testing the potency of BAL-0028. Cells were plated at a density of 40,000 cells/well in KBM Gold Keratinocyte Growth Basal Medium (Lonza) supplemented with KGM Gold Keratinocyte Growth Medium SingleQuots Supplements and Growth Factors (Lonza) and 0.03 µM calcium carbonate (CaCl_2_; Sigma-Aldrich). After overnight incubation at 37°C and 5% CO_2_, differentiation of NHEKs was induced by increasing the concentration of CaCl_2_ to 1.5 mM, and cells were incubated for a further 24 h. Talabostat (MedChem Express; final concentration 10 µM) was used to activate NLRP1, and cells were incubated overnight with the agonist in the presence or absence of BAL-0028 or MCC950, 10-fold concentrations ranging from 10 nM to 10 µM. Supernatants were collected for cytokine analysis. The caspase-1 inhibitor, VX-765 (InvivoGen; final concentration 10 µM), was used as a positive control for inflammasome inhibition.

### Mouse J774A.1 NLRP3 inflammasome activation assay

J774A.1 cells (ATCC) were cultured in complete growth media composed of DMEM high glucose (Gibco)/10% FBS (Corning)/pen/strep (Caisson). Cells were spun down and resuspended to 500,000 cells/ml in GM-2. 100,000 cells (200 μl) were then added to each well of a 96-well TC plate and incubated O/N in a standard cell culture incubator (37°C; 5% CO_2_). After this incubation, the plate was tilted, and the media were carefully removed. 200 μl of GM-2 or GM-2 containing 100 ng/ml LPS (*E. coli* O26:B6; Sigma-Aldrich) was then added to the appropriate wells, and the cells were incubated for an additional 5 h. The media was again removed and replaced with Opti-Mem medium (without serum; Thermo Fisher Scientific) containing predetermined dilutions of test compounds in replicate wells. After a 30-min incubation, 10 µM nigericin (Sigma-Aldrich) in Opti-Mem medium with the corresponding concentration of test compound was added to the wells for 1 h. Positive control wells contain 10 µM nigericin in Opti-Mem in the absence of test compound, while negative control wells contain Opti-Mem only. Supernatants were then transferred to a 96-well plate for storage and assayed for IL-1β (human; DuoSet; R&D) and relative LDH levels using a CytoTox 96 Kit (Promega). Once supernatants were removed, the relative viability of adherent cells in the 96-well TC plate was determined using a CellTiter-Glo luminescent cell viability assay (Promega).

### CD14^+^ (human, canine, and monkey) and PBMCs (minipig, rabbit, and rat) NLRP3 inflammasome activation assays

Human CD14^+^ monocytes were freshly isolated from ∼30 ml of heparinized whole blood collected by venipuncture from healthy human volunteers and collected into heparin tubes from each donor. PBMCs were isolated from whole blood using Sepmate (cat. no. 85450; STEMCELL Technologies) tubes, following the manufacturer’s recommended protocol. PBMCs were then magnetically sorted to enrich CD14^+^ cells using microbeads conjugated to a monoclonal human CD14 antibody (cat. no. 130-050-201; Miltenyi Biotec). Cryopreserved Beagle canine CD14^+^, cynomolgus monkey CD14^+^ cells, New Zealand white rabbit PBMCs, and Wistar rat PBMCs were obtained from IQ Biosciences. Cryopreserved Gottingen minipig PBMCs were obtained from BioIVT. African green monkey (AGM-1) CD14^+^ cells were isolated from cryopreserved PBMCs (Virscio) using nonhuman primate CD14^+^ MACS MicroBeads and the supplied MACS separation protocol (Miltenyi). AGM-1 CD14^+^ cells were isolated immediately upon thawing of the PBMCs. Upon thaw, cells were spun down and resuspended to 500,000–700,000 cells/ml in pre-warmed complete growth composed of RPMI-1640 + GlutaMAX (Gibco)/10% FBS (Corning)/pen/strep (Caisson). 50,000–70,000 cells (100 μl) were then added to the wells of a 96-well TC plate, and the cells were incubated for 1 h in a standard cell culture incubator (37°C; 5% CO_2_) to recover. 100 μl of GM-3 containing 200 ng/ml LPS (*E. coli* O26:B6; Sigma-Aldrich) was then added to the wells (100 ng/ml final concentration), and the cells were incubated for 5 h. The media was again removed and replaced with Opti-Mem medium (without serum; Thermo Fisher Scientific) containing predetermined dilutions of test compounds in replicate wells. After a 30-min incubation, 10 µM nigericin (final concentration; Sigma-Aldrich) in Opti-Mem medium with the corresponding concentration of test compound was added to the wells for an additional 1 h. Positive control wells contain 10 µM nigericin in Opti-Mem in the absence of test compound, while negative control wells contain Opti-Mem only. Supernatants are then transferred to a fresh 96-well plate for storage and assayed for IL-1β (canine, monkey, porcine, rabbit, or rat; DuoSet; R&D) and relative LDH levels using a CytoTox 96 Kit (Promega). Once supernatants are removed, the relative viability of adherent cells in the 96-well TC plate is determined using a CellTiter-Glo luminescent cell viability assay (Promega).

### Immortalized BMDM NLRP3 inflammasome activation assays

Generation of 129S6 human promoter-*NLRP3* mice has been previously described (Snouwaert et al., 2016). In brief, tibias and femurs from WT 129S6 or 129S6 human promoter-*NLRP3* male mice were removed, and the bone marrow was harvested by flushing with fresh medium. Bone marrow was plated in BMDM medium: DMEM high glucose (Gibco), 10% FBS (Gibco), 100 mM sodium pyruvate (Gibco), and 50 U pen/strep (Gibco), supplemented with 100 ng/ml human M-CSF (Proteintech). Cells were incubated in a standard cell culture incubator (37°C; 5% CO_2_) for 5 days. Media were removed and replaced with media containing the J2 CRE virus (carrying v-myc and v-Raf/v-Mil oncogenes, kindly donated by Dr Joana Sá-Pessoa, Queen’s University Belfast) for 24 h before being replaced with fresh BMDM medium containing M-CSF. Cells were continuously cultured for 9 wk with a gradual reduction in M-CSF until cells doubled every 24 h in M-CSF–free media. Immortalized BMDM (iBMDM) were plated at 0.5 × 10^6^/ml in 96-well TC plates in 100 μl BMDM medium (no M-CSF). For NLRP3 assays, the following day the media was removed and replaced with Opti-Mem ±100 ng/ml Ultrapure LPS from *E. coli* K12 (InvivoGen) for 3.5 h. Inhibitors were prepared by serial dilution in DMSO. The media were removed and replaced with Opti-Mem containing test compounds or 0.1% DMSO vehicle control. After 30-min incubation, 5 μM nigericin (Adipogen) was added for 1 h. Cell-free supernatants were removed and assessed by LDH assay (Roche) and ELISA for IL-1β according to the manufacturer’s instructions (mouse DuoSet, R&D Systems).

### Peritoneal macrophage NLRP3 inflammasome activation assays

The generation of 129S6 human promoter-NLRP3 and 129S6 mouse promoter-NLRP3 mice has been described previously (Koller et al., 2024; Snouwaert et al., 2016). Peritoneal macrophages were isolated from 3-mo-old male 129S6-WT or 129S6-human promoter or mouse promoter *NLRP3* mice by lavage with 3–5 ml of PBS (no Ca^2+^ and no Mg^2+^). Cells from two to four individual mice were combined for each experiment. Fresh cells were then spun down and resuspended in complete growth media RPMI-1640 + GlutaMAX (Gibco)/10% FBS (Corning)/pen/strep (Caisson), and 50,000–100,000 cells (150 μl) were then added to the appropriate wells of a 96-well TC plate. The cells were incubated overnight in a standard cell culture incubator (37°C; 5% CO_2_). The following day 50 μl of GM-1 containing 400 ng/ml LPS (*E. coli* O26:B6; Sigma-Aldrich) was added to the appropriate wells (final concentration; 100 ng/ml LPS), and the cells were incubated for 5 h. The media was removed and replaced with Opti-Mem medium (without serum; Thermo Fisher Scientific) containing predetermined dilutions of test compounds in replicate wells. After a 30-min incubation, 10 µM nigericin (final concentration; Sigma-Aldrich) or 4 mM ATP (final concentration; TOCRIS) in Opti-Mem medium with the corresponding concentration of test compound was added to the wells for an additional 1 h. Positive control wells contain 10 µM nigericin or 4 mM ATP in Opti-Mem in the absence of test compound, while negative control wells contain Opti-Mem only. Supernatants are then transferred to a fresh 96-well plate for storage and assayed for IL-1β (DY401-05; mouse; DuoSet; R&D) and/or relative LDH levels (as a surrogate for pyroptosis) using a CytoTox 96 Kit (G1780; Promega).

### Sequence alignments

Multiple protein sequence alignments were performed using C*LUSTAL W* Multiple Sequence Alignment Program (version 1.83). Protein sequences from the following GenBank accession numbers were used: *Homo sapien* (human) NLRP3 Q96P20.3, *M. fascicularis* (cynomolgus monkey; crab-eating macaque) NLRP3 XP_045246555, *C. sabaeus* (African green monkey) NLRP3 XM_007990053.2, *Mus musculus* (mouse) NLRP3 NP_001346567.1, *Rattus norvegicus* (rat) NLRP3 NM_001191642.1, *Oryctolagus cuniculus* (European rabbit) NLRP3 QHZ00929.1, and *Canis lupus familiaris* (dog) NLRP3 [transcript variant X1] XM_038673023.1. For all species, the sequence alignments were restricted to the corresponding sequence of amino acids 131–694 in the human NACHT domain construct used in nanoDSF studies (Fig. S4 C).

### ADP-Glo kinase assay

To assess NLRP3 ATPase activity, the ADP-Glo Kinase Assay (Promega) was used according to the manufacturer’s supplied instructions. Specific assay conditions were as follows: 2.5 µM of MBP-ΔNLRP3-HIS protein construct (Hartman et al., 2024) or MBP-NLRP3 (3-1036) peak 1 protein (Brinkschulte et al., 2022) was preincubated with predetermined dilutions of test compounds for 15 min at 37°C prior to the addition of 25 µM of UltraPure ATP (Promega) for 2 h at 37°C. For the MBP-ΔNLRP3-HIS protein, the assay buffer was composed of 50 mM Tris, pH 7.5, 150 mM NaCl, 10 mM MgCl_2_, 10% glycerol, and 0.005% Tween-20. For MBP-NLRP3 (3-1036) peak 1 protein, the assay buffer was composed of 50 mM HEPES, pH 7.5, 150 mM NaCl, 10 mM MgCl_2_ and 0.2 mM tris(2-carboxyethyl) phosphine. Replicate wells of each condition were tested in two to three separate experiments. Luminescence was recorded with an integration time of 100 ms on either a Tecan Infinite M1000 PRO, Molecular Devices SpectraMax M5e, or on a PHERAstar FSX with an integration time of 1 s.

### nanoDSF assays

For the noncompetitive thermal shift assay, 3 µM NLRP3^NACHT^ protein was incubated for 30 min on ice with 2% DMSO, 10 µM BAL-0028, 10 μM MCC950, or BAL-0598 (1:3.3 ratio of protein to compound). The assay buffer contained 50 mM HEPES, pH 7.5, 150 mM NaCl, 10 mM MgCl_2_, 5 mM β-mercaptoethanol, 150 mM L-arginine, and 1 mM ADP. For the competitive thermal shift assay, 3 µM NLRP3^NACHT^ protein was treated with 2% DMSO as the reference, or various concentrations of MCC950 and BAL-0028. Treatment of 3 µM NLRP3^NACHT^ protein with 20 μM MCC950 was compared with 10 μM MCC950 plus 10 µM BAL-0028, and treatment with 40 μM MCC950 was compared with 30 μM MCC950 plus 10 µM BAL-0028. The measurements were set up with a temperature ramp ranging from 20 to 90°C, and a slope of 1.5°C per minute, and at 100% laser intensity. Data points are an average of two technical replicates from three independent experiments.

### SPR experiments

The binding of BAL-0028 and BAL-0598 to human NLRP3 was studied by SPR spectroscopy. Therefore, Avi-MBP–tagged human NLRP3^NACHT^ protein, biotinylated during *Sf9* insect cell expression by coexpression with BirA, was immobilized on a streptavidin sensor chip (Cytiva), docked in a Biacore 8K instrument (GE Healthcare). All SPR experiments were conducted at 25°C. A running buffer of 10 mM HEPES pH 7.4, 200 mM NaCl, 0.5 mM ADP, 0.5 mM tris(2-carboxyethyl) phosphine, 2 mM MgCl_2_, 1 g/liter carboxymethyl dextran hydrogel (CMD), 0.05 % Tween-20, and 2 % DMSO was used.

After equilibration of the NLRP3^NACHT^ sensor surface (2 h at a flow rate of 100 μl/min), BAL compounds were tested in single-cycle kinetics mode (flow rate: 30 μl/min, association phase: 240 s, dissociation phase: 600 s), injecting a fivefold dilution series (highest concentration: 3 µM). Data were collected at 10 Hz and corrected by a four-point DMSO solvent correction as well as double referenced by reference flow cell subtraction and blank cycle. For determining the kinetic rate constants (*k*_a_ and *k*_d_) and the dissociation constant *K*_D_, the data were fitted to a 1:1 interaction model (*K*_D_ [1:1 binding]) or a steady-state model (*K*_D_ [steady state]), respectively, using the Biacore Insight Evaluation Software (version 6.0.7.1750, Cytiva).

### DARTS assay

The DARTS assay was adapted from a published protocol (Pai et al., 2015). HEK293T cells (Merck) were cultured in DMEM + 10% FCS + 50 U pen/strep + 1% Glutamax (all from Gibco). Cells were seeded at 3 × 10^5^/ml in 10-cm^2^ dishes. The next day they were transfected with 3–5 μg of plasmids, pEF6_human NLRP3-mCherry (a kind gift from Prof. Kate Schroder, University of Queensland, Brisbane, Australia), pCDNA3.4 human NLRP3-Twin-Strep-tag, or human NLRP3_1-688-Twin-Strep-tag (synthesized by GenScript) using the calcium phosphate method (Kwon and Firestein, 2013). Before cells were harvested, 0.1–10 μM BAL-0028 or MCC950 or DMSO vehicle control was added. 24 h after transfection, cells were harvested. Media were removed, and cells were washed in PBS containing inhibitors or DMSO control; cells were lysed in lysis buffer (50 mM Tris-HCl, pH 7.4, 150 mM NaCl, 1 mM ATP, 2 mM EDTA, and 0.5% Igepal CA-630) containing protease inhibitors (complete mini protease inhibitor cocktail; Roche), benzonase, and inhibitors or DMSO as indicated. Lysates were disrupted by passage through a 27-gauge needle and cleared by centrifugation at 14,000 *g* for 10 min at 4°C. Protein concentration was determined using a Pierce Rapid Gold BCA Protein Assay Kit (Thermo Fisher Scientific). Pronase and thermolysin 10 mg/ml, (Merck) were added at the indicated protease-to-protein ratio for 15 min at room temperature. The reaction was stopped by the addition of 20× protease inhibitor cocktail and incubated on ice for 10 min.

Protein samples were prepared with NuPAGE LDS sample buffer (Thermo Fisher Scientific) supplemented with 10 mM DTT. Samples were then resolved by SDS-PAGE using 4–20% Mini-PROTEAN TGX stain-free gels (BioRad) and transferred onto nitrocellulose membrane using the Trans-Blot Turbo transfer system (BioRad). Membranes were blocked in 5% (wt/vol) dried milk in TBS-T (10 mM Tris/HCl, pH 8, 150 mM NaCl, and 0.05% (vol/vol) Tween-20) for 1 h at room temperature. Membranes were incubated with primary antibody NLRP3 clone D4D8T at 1:1,000 (15101; Cell Signaling Technology) diluted in 5% (wt/vol) dried milk in TBS-T and then with Peroxidase-AffiniPure Goat Anti-Rabbit IgG (Jackson ImmunoResearch) at 1 in 5,000. Membranes were developed using Clarity Western ECL substrate (BioRad) and then visualized using a Syngene G:Box.

### Mouse plasma protein binding determination

On the day of the experiment, pooled male CD-1 mouse EDTA-K^2^ plasma (BIOIVT) was thawed and centrifuged at 3,220 rpm for 5 min, and only plasma within the range of pH 7.0–8.0 was used. Working solutions of BAL-0598 and the control compound warfarin were prepared, and aliquots of working solutions were spiked into a blank plasma matrix (10—99.5%) to achieve a final concentration of 2, 10, 30, or 100 µM of BAL-0598. The HT-Dialysis plate and the dialysis membranes (molecular weight cutoff 12–14 KDa) were purchased from HT Dialysis LLC (Gales Ferry, CT). The dialysis buffer was composed of 100 mM sodium phosphate and 150 mM NaCl, pH 7.4 ± 0.1), and the stop solution was composed of acetonitrile containing 200 ng/ml tolbutamide and 200 ng/ml labetalol. Dialysis membranes were pre-treated according to the manufacturer’s instructions, and the dialysis instrument was prepared according to the manufacturer’s instructions. For T0 samples, aliquots of mouse plasma containing BAL-0598 or control compound were transferred in triplicate to the sample collection plate and mixed with blank buffer at 1:1 (vol:vol). Stop solution was then added, and the plate was sealed and mixed at 800 rpm for 10 min and stored at 2–8°C until all samples were ready for analysis. Ultracentrifugation was performed with a Beckman Coulter, OptimaTM L-90K. To prepare protein-free samples (F samples) for unbound determination, an aliquot of the preincubated matrix containing BAL-0598 or control compound was transferred to ultracentrifuge tubes (*n* = 2) and subjected to ultracentrifugation at 37°C, 155,000 × *g* (35,000 rpm) for 4 h. At the end of the ultracentrifugation, F sample aliquots were taken from the second layer down in the supernatant. To prepare the T4.5 samples, the residual aliquot of preincubated spiked plasma was placed into the incubator for 4 h. These samples were then transferred to new 96-well plates, and an equal volume of buffer or plasma was added to each well. Stop solution was then added to these samples, and the mixture was vortexed and centrifuged at 4,000 rpm for about 20 min. After dialysis or ultracentrifugation, compound levels were determined by LC-MS/MS analysis. Dialysis: %Unbound = 100 × F/T; %Bound = 100 − %Unbound; %Recovery = 100 × (F + T)/T0; where F = free compound concentration as determined by the calculated concentration on the buffer side of the membrane, T = total compound concentration as determined by the calculated concentration on the matrix side of the membrane, and T0 = total compound concentration as determined by the calculated concentration in matrix before dialysis. Ultracentrifugation: %Unbound = 100 × F/Mean of T4.5; %Bound = 100 − %Unbound; %Remaining = 100 × Mean T4.5/Mean of T0.

### *In vivo* LPS/ATP-induced peritonitis mouse model

All experiments were approved by the Institutional Animal Care and Use Committee of BioAge Labs. Female 4-mo-old 129S6-hNLRP3 mice were group housed during acclimation before the experiment and randomized to different treatment group cages on the day of the experiment. Mice were housed conventionally in a constant temperature (18–26°C) and humidity (30–70%) animal room with a 12/12 h light/dark cycle and free access to food (5lG4; LabDiet JL Rat and Mouse/Irr 6F) and water (M-WB-300A; Innovive) *ad libitum*. Temperature and relative humidity were monitored daily. The animals were identified by cage card and tail mark during the study to identify the animal individually.

In the peritonitis studies, the mice were i.p. injected with LPS at 40 µg/kg, followed 2 h later by an i.p. injection of ATP disodium salt at 304.4 mg/kg. Animals not treated with ATP (i.e., group 1) were dosed with the appropriate volume of PBS. BAL-0598 was administered to feed mice once at 3, 10, 30, or 100 mg/kg by oral gavage (PO) at 10 μl/g following facility standard operating procedures. Vehicle (20% propylene glycol + 40% PEG-400 + 10% ethanol) only or BAL-0598 was PO dosed 1 h prior to ATP i.p. administration, which approximately corresponded to the plasma T_max_ of BAL-0598 (data not shown) at the time of ATP administration. 30 min after ATP administration, submental bleeds were collected in a blood collection tube with EDTA-K^2^ (BD 365974). Blood samples were centrifuged at ∼4°C, 13,000 × *g* for 10 min to collect plasma to determine total plasma BAL-0598 concentrations by LC-MS/MS (Quintara Discovery), and then the mice were immediately euthanized with isoflurane, and the peritoneal cavity was lavaged with 3 ml of PBS to collect the PLF. Plasma BAL-0598 levels were determined by LC-MS/MS. The level of mature IL-1β and IL-6 in the PLF was determined by ELISA (R&D Systems).

### *In vivo* IC_50_ determination

For *in vivo* IC_50_ determinations, the absolute values of IL-1β in the PLF were plotted against the unbound BAL-0598 concentrations (nM) determined at the time of euthanasia (i.e., 1.5 h after BAL-0598 or vehicle dosing). Unbound BAL-0598 values were calculated from the total BAL-0598 measured in the plasma of individual mice using an unbound value of 1.61% (Table S1). Relative IC_50_ values (i.e., the unbound BAL-0598 concentration required to bring the curve down to a point halfway between the “top” and “bottom” plateaus of the curve) were calculated by nonlinear regression analysis using GraphPad PRISM version 10.1.1. The upper plateaus were anchored using the IL-1β values derived from the vehicle-dosed mouse promoter 129S6-hNLRP3 mice treated with LPS + ATP, with the unbound BAL-0598 set to a default value of 0.002 nM (one order of magnitude below the lower limit of quantification).

### Primary NLRP3-AID study subjects, blood sample acquisition, PBMC isolation, and stimulation

NLRP3-AID patients with verified NLRP3 gain-of-function mutations (Y570C, A354V, T348M, and A439V) were recruited at the Department of Pediatrics, University Hospital Tübingen, and healthy blood donors at the Institute of Immunology, Department of Innate Immunity, University of Tübingen. All patients and healthy blood donors included in this study provided their written informed consent before study participation. Approval for use of their biomaterials was obtained by the ethics commission of the Medical Faculty Tübingen (approval number 795/2017BO1), in accordance with the principles laid down in the Declaration of Helsinki as well as applicable laws and regulations.

PBMCs from healthy donors and patients were isolated from whole blood using Ficoll density gradient purification and frozen in 10% DMSO-freezing media in liquid nitrogen. For analysis, cells were thawed and, after an overnight rest in RPMI1640 media with 10% FCS primed with 10 ng/ml LPS for 3 h, treated with MCC950 and BAL-0028 (200 and 1,000 nM) for 30 min before stimulation with 10 µM nigericin for 1 h in biological triplicate. Supernatants were then collected for triplicate ELISA according to the manufacturer’s instructions (BioLegend Human IL-1β ELISA Kit).

### U937 expressing NLRP3-AID–associated mutant assays

NLRP3-deficient U937 cells reconstituted with doxycycline-inducible NLRP3 mutants have been previously described (Cosson et al., 2024). 0.3 × 10^6^ U937 cells/ml were platted in RPMI 1,640 GlutaMax-I supplemented with 1× PS and 10% FBS (Gibco). The next day, cells were treated with BAL-0028, BAL-0598, or MCC950 (1,000, 200, and 40 nM, with a final DMSO concentration of 0.05% in all conditions). 15 min later, cells were treated with doxycycline (1 μg/ml, 3 h, Sigma-Aldrich), LPS (40 ng/ml, 2 h, O111:B4, Sigma-Aldrich), and nigericin (15 μg/ml; InvivoGen) before time-lapse imaging using a CQ1 high-content screening microscope (Yokogawa). PI (1.25 μg/ml) and Hoechst (0.2 μg/ml) were added 2 h before imaging. 2 images/well were taken every 15 min for 2 h using 10X objectives (UPLSAPO 10X/0.4). Image analysis was performed as previously described (Cosson et al., 2024).

### Data analyses

The absolute values of IL-1β in the cell culture supernatants were normalized relative to the mean level of IL-1β present in the positive control (i.e., the maximum response) and shown as a percent of maximum response. These values were calculated independently within each experiment. Relative IC_50_ values (i.e., the BAL-0028 or MCC950 concentration required to bring the curve down to a point halfway between the top and bottom plateaus of the curve) were calculated by nonlinear regression analysis using GraphPad PRISM version 9.5.0.

For cytotoxicity (LDH release) calculations, raw absorbance values at 490 nm (OD_490_) of cell culture supernatants were normalized relative to the OD_490_ of the maximal LDH release control (10× Lysis Reagent). Percent cytotoxicity was calculated using the formula $\%\text{Cytotoxicity}=100\times \left(\text{Experimental}\;\text{LDH}\;\text{release}\;\left({\text{OD}}_{490}\right)/\text{Maximal}\;\text{LDH}\;\text{release}\;\left({\text{OD}}_{490}\right)\right).$

### Online supplemental material

Fig. S1 shows that BAL-0028 does not inhibit inflammasome-independent cytokine release and that it is not cytotoxic. Fig. S2 shows that BAL-0028 does not inhibit AIM2- or NAIP/NLRC4-dependent pyroptosis. Fig. S3 shows that BAL-0028 inhibits human NLRP3 in mouse cells, and it also shows a multiple sequence alignment of NLRP3^NACHT^ highlighting amino acid differences between species. Fig. S4 shows that BAL-0028 stabilizes NLRP3 in DARTS assays and that it does not synergize with MCC950 for NLRP3 inhibition. Fig. S5 shows that BAL-0598 inhibits the activation of human and monkey NLRP3 and that it is a non-covalent inhibitor. Table S1 shows the binding of BAL-0028 and BAL-0598 to mouse plasma protein.

## Supplementary Material

Table S1 shows the mouse plasma protein binding of BAL-0028 and BAL-0598.

SourceData F2 is the source file for Fig. 2.

SourceData FS4 is the source file for Fig. S4.

## Data Availability

All data reported in this paper will be shared by the corresponding authors (R.C. Coll and K. Wilhelmsen) upon reasonable request.
