# Supplementary material for: Discovery of potent and selective inhibitors of human NLRP3 with a novel mechanism of action
Source: J Exp Med. 2025 Sep 2;222(11):e20242403. doi: 10.1084/jem.20242403 (PMC12404154; doi:10.1084/jem.20242403)
Supplement: Table S1 — shows the mouse plasma protein binding of BAL-0028 and BAL-0598. [file jem_20242403_tables1.docx]

**Table S1. Mouse plasma protein binding of BAL-0028 and BAL-0598**

| **BAL-0028** |  |  |  |  |
| --- | --- | --- | --- | --- |
| **Compound (µM)** | **Plasma (%)** | **Unbound (%)** | **Bound (%)** | **Recovery (%)** |
| 2 | 99.5 | 0.11 | 99.89 | 87.53 |
| 2 | 10 | 0.10 | 99.90 | 96.13 |
| 2 | 20 | 0.12 | 99.88 | 86.53 |
| **BAL-0598** |  |  |  |  |
| **Compound (µM)** | **Plasma (%)** | **Unbound (%)** | **Bound (%)** | **Recovery (%)** |
| 2 | 99.5 | 1.84 | 98.16 | 98.57 |
| 2 | 99.5 | 0.76 | 99.24 | 89.77 |
| 2 | 99.5 | 1.42 | 98.58 | 90.78 |
| 10 | 99.5 | 1.70 | 98.30 | 96.06 |
| 30 | 99.5 | 1.69 | 98.31 | 97.87 |
| 100 | 99.0 | 2.24 | 97.76 | 86.94 |
