# Supplementary material for: Discovery of potent and selective inhibitors of human NLRP3 with a novel mechanism of action
Source: J Exp Med. 2025 Sep 2;222(11):e20242403. doi: 10.1084/jem.20242403 (PMC12404154; doi:10.1084/jem.20242403)
Supplement: SourceData F2 — is the source file for Fig. 2. [file jem_20242403_sourcedataf2.pdf]

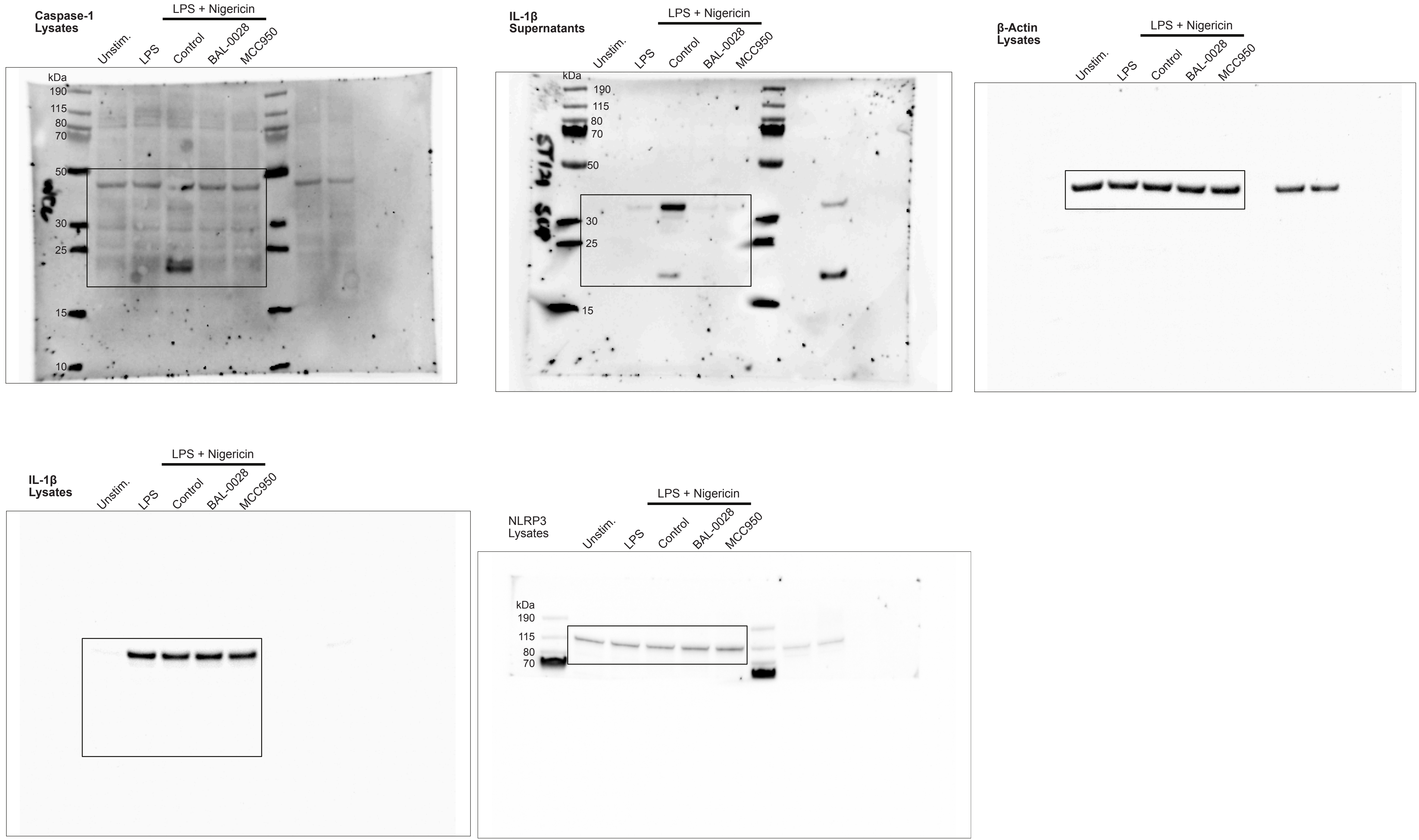
Caspase-1 Lysates
LPS + Nigericin
Unstim.
LPS
Control
BAL-0028
MCC950
kDa
190
115
80
70
50
30
25
15
10
IL-1β Supernatants
LPS + Nigericin
Unstim.
LPS
Control
BAL-0028
MCC950
kDa
190
115
80
70
50
30
25
15
β-Actin Lysates
LPS + Nigericin
Unstim.
LPS
Control
BAL-0028
MCC950
IL-1β Lysates
LPS + Nigericin
Unstim.
LPS
Control
BAL-0028
MCC950
NLRP3 Lysates
LPS + Nigericin
Unstim.
LPS
Control
BAL-0028
MCC950
kDa
190
115
80
70
