# Supplementary material for: Discovery of potent and selective inhibitors of human NLRP3 with a novel mechanism of action
Source: J Exp Med. 2025 Sep 2;222(11):e20242403. doi: 10.1084/jem.20242403 (PMC12404154; doi:10.1084/jem.20242403)
Supplement: SourceData FS4 — is the source file for Fig. S4. [file jem_20242403_sourcedatafs4.pdf]

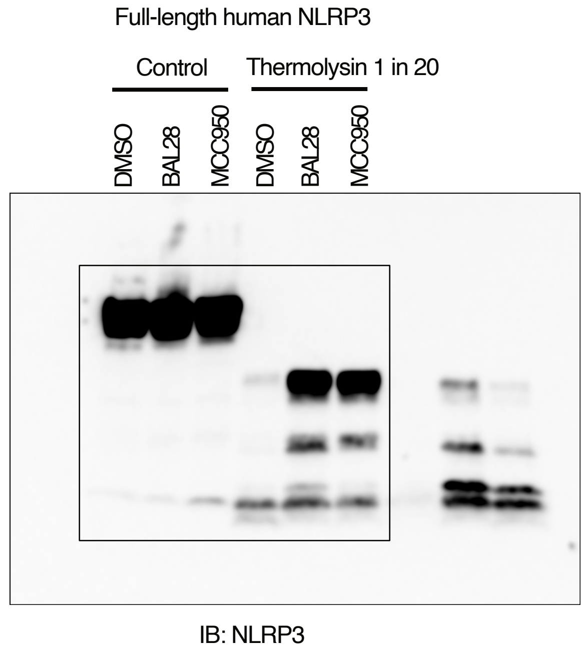
Full-length human NLRP3
Control
Thermolysin 1 in 20
DMSO
BAL28
MCC950
DMSO
BAL28
MCC950
IB: NLRP3

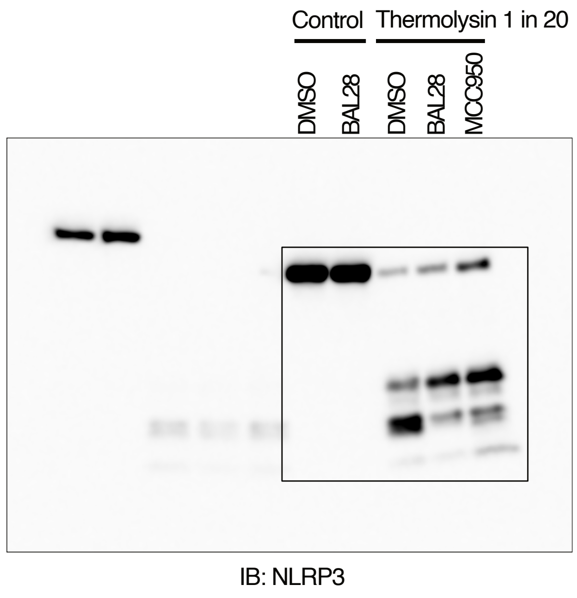
Control
Thermolysin 1 in 20
DMSO
BAL28
DMSO
BAL28
MCC950
IB: NLRP3

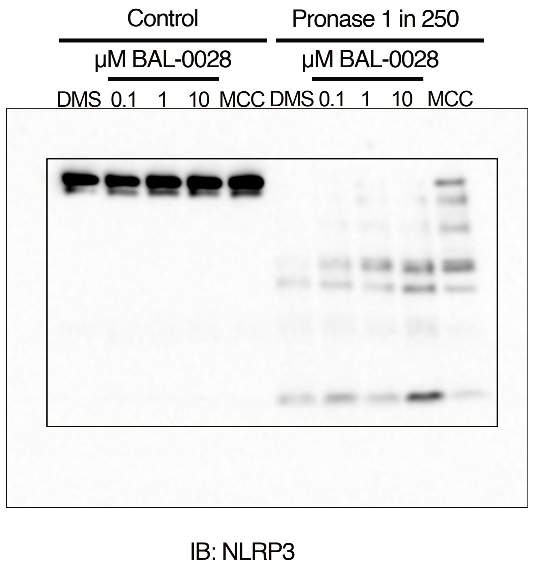
Control
Pronase 1 in 250
μM BAL-0028
μM BAL-0028
DMS
0.1
1
10
MCC
DMS
0.1
1
10
MCC
IB: NLRP3
